# A complete set of cross-correlated relaxation experiments for determining the protein backbone dihedral angles

**DOI:** 10.1007/s10858-025-00458-x

**Published:** 2025-03-20

**Authors:** Paulina Bartosińska-Marzec, Bartłomiej Banaś, Clemens Kauffmann, Andreas Beier, Daniel Braun, Irene Ceccolini, Wiktor Koźmiński, Robert Konrat, Anna Zawadzka-Kazimierczuk

**Affiliations:** 1 Biological and Chemical Research Centre, Faculty of Chemistry, University of Warsaw, Żwirki i Wigury 101, 02-089 Warsaw, Poland; 2 Max Perutz Laboratories, Department of Structural and Computational Biology, University of Vienna, Vienna Biocenter Campus 5, 1030 Vienna, Austria; 3 Wiener Linien GmbH and Co KG, Erdbergstraße 202, 1030 Vienna, Austria

**Keywords:** Cross-correlated relaxation, Dihedral angles distribution, Protein structural propensities, Multidimensional NMR, Intrinsically disordered proteins

## Abstract

The investigation of structural propensities of proteins is essential for understanding how they function at the molecular level. NMR, offering atomic-scale information, is often the method of choice. One of the available techniques relies on the cross-correlated relaxation (CCR) effect, whose magnitude is related to local spatial conformation. Application of these methods is difficult if the protein under investigation exhibits high mobility, because NMR observables like CCR rates and chemical shifts present themselves as mere averages of an underlying ensemble distribution. Furthermore, relaxation observables are a convolution of structural and dynamical components. Despite these challenges, it is possible to infer the underlying structural ensemble by combining information from several CCR rates with a different geometrical dependence. In this paper, we present a set of eight CCR experiments tailored for proteins of a highly dynamic nature. Analyzed together, they yield a distribution of backbone dihedral angles for each residue of the protein. The experiments were validated on the folded protein ubiquitin using PDB-deposited NMR structures for comparison. Extraordinary peak separation, achieved by evolving four different chemical shifts, allows for the application of this method to intrinsically disordered proteins in future studies.

**Supplementary Information:**

The online version contains supplementary material available at 10.1007/s10858-025-00458-x.

## Introduction

NMR offers a plethora of methods for structural studies of biomolecules. One such method is based on the phenomenon of interference between different relaxation mechanisms. This phenomenon is called cross-correlated relaxation (CCR) effect. For diamagnetic proteins in solution state, the most efficient relaxation mechanisms are dipolar interaction (DD) and chemical shift anisotropy (CSA), which can interfere with each other. For macromolecules in solution and at high magnetic field, the CCR rate for a given pair of interactions *u* and *v*, $\varGamma _{u,v}$, is proportional to the spectral density at zero frequency (Brutscher 2000):

1 $$\begin{aligned} \varGamma _{u,v} \propto J_{u,v}(0)=\int _{0}^{\infty }C_{u,v}(t) dt \end{aligned}$$

where $C_{u,v}$ is a corresponding time correlation function. The form of the time correlation function is a result of the stochastic motions of the unit vectors ${\textbf {u}}$ and ${\textbf {v}}$ corresponding to the involved interactions (see below for details), which occur in time *t* as a result of the protein dynamics. Importantly, the dynamics can be of any type, no particular motion type is postulated at this stage. The only assumption is that the distance between nuclei involved in dipolar interactions is constant. The distance dependence is introduced below (see Eqs. 9 and 10). The time correlation function can be expressed as follows (Daragan and Mayo 1997):

2 $$\begin{aligned} C_{u,v}(t)=\bigl \langle P_2({\textbf {u}}(0)\cdot {\textbf {v}}(t)) \bigr \rangle \end{aligned}$$

where $P_{2}$ is the second-order Legendre polynomial $P_2(x)=\frac{1}{2}(3x^2-1)$ and angled brackets denote the average over the ensemble of protein molecules in the sample. By combining the Eqs. 1 and 2, it is possible to express the spectral density at zero frequency in a form showing the geometrical and dynamical contributions:

3 $$\begin{aligned} J(0)&=C_{u,v}(0)\int _{0}^{\infty }\frac{C_{u,v}(t)}{C_{u,v}(0)}dt=\\ &=\bigl \langle P_2({\textbf {u}}(0)\cdot {\textbf {v}}(0)) \bigr \rangle \nonumber \int _{0}^{\infty }\frac{C_{u,v}(t)}{C_{u,v}(0)}dt=\\&=\bigl \langle P_2(\cos \theta _{uv}) \bigr \rangle \tau \end{aligned}$$

where $\theta _{uv}$ is the angle between the unit vectors *u* and *v* at time $t=0$, and $\tau$, which is equal to the integral of the normalized time correlation function, is called correlation time.

Therefore, both the geometry ($\langle P_2(\cos \theta _{uv}) \rangle$) and the dynamics ($\tau$) of the system under investigation contribute to the CCR effect. For CCR rates involving protein backbone nuclei, assuming the standard geometry of peptide bonds and the orientation of the CSA tensor principal axes (Cisnetti et al. 2004), the angle between the ${\textbf {u}}$ and ${\textbf {v}}$ vectors is directly related to the protein dihedral angles: $\theta _{uv}=\theta _{uv}(\phi , \psi )$, and consequently the CCR rates can be the source of protein structural information. The geometrical contribution, being averaged over the molecular ensemble, in which each backbone conformation ($\phi$, $\psi$) is adopted with a certain probability $p_{\phi ,\psi }$, can be expressed as:

4 $$\begin{aligned} \bigl \langle P_2(\cos \theta _{uv}(\phi ,\psi )) \bigr \rangle = \sum _{\phi ,\psi }p_{\phi ,\psi }P_2(\cos \theta _{u,v}(\phi ,\psi )) \end{aligned}$$

The overall form of the CCR rate contains the three following factors:

5 $$\begin{aligned} \varGamma = PreFactor \cdot DynamicFactor \cdot GeomFactor \end{aligned}$$

The *PreFactor* depends on the relaxation mechanisms involved (Brutscher 2000), that is:

for DD-DD interference (AB and CD dipole pairs):

6 $$\begin{aligned} PreFactor_{DD-DD} = \frac{2}{5} \left( \frac{\mu _{0} \hbar }{4\pi }\right) ^{2}\gamma _{A} \gamma _{B}\gamma _{C} \gamma _{D} \end{aligned}$$

for DD-CSA interference (AB dipole pair and E nucleus CSA):

7 $$\begin{aligned} PreFactor_{DD-CSA} = \frac{4}{15} \frac{\mu _{0} \hbar }{4 \pi } \gamma _{A} \gamma _{B} \gamma _{E} B_{0} \end{aligned}$$

for CSA-CSA interference (E and F nuclei CSA):

8 $$\begin{aligned} PreFactor_{CSA-CSA} = \frac{8}{45} \gamma _{E} \gamma _{F} B_{0}^{2} \end{aligned}$$

where $\mu _{0}$ is the vacuum permeability, $\hbar$ is the reduced Planck constant, $\gamma _{i}$ is the gyromagnetic ratio of i nucleus, and $B_{0}$ is the magnetic field strength.

The *DynamicFactor* is the weighted average correlation time $\tau$, and the *GeomFactor* is derived from the relative positions and orientations of the vectors describing the relaxation mechanisms involved (Yang et al. 1997). For dipolar interaction, the vector points from one of the nuclei involved to another one; for CSA, we derive the vector from the principal components of the CSA tensor.

Therefore, for DD–DD, we get:

9 $$\begin{aligned} \begin{aligned}&GeomFactor_{DD-DD} =\\&=\Bigl \langle \frac{1}{r_{AB}^{3}} \cdot \frac{1}{r_{CD}^{3}} \cdot P_2(cos\measuredangle (\vec {r}_{AB},\vec {r}_{CD})) \Bigr \rangle \end{aligned} \end{aligned}$$

For DD–CSA, we get:

10 $$\begin{aligned} \begin{aligned}&GeomFactor_{DD-CSA} =\\&=\Bigl \langle \frac{1}{r_{AB}^{3}} \cdot \Big ((\sigma ^{E}_{xx}-\sigma ^{E}_{zz}) \cdot P_2(cos\measuredangle (\vec {X}^{E},\vec {r}_{AB})) +\\&+(\sigma ^{E}_{yy}-\sigma ^{E}_{zz}) \cdot P_2(cos\measuredangle (\vec {Y}^{E},\vec {r}_{AB}))\Big ) \Bigr \rangle \end{aligned} \end{aligned}$$

For CSA–CSA, we get:

11 $$\begin{aligned} \begin{aligned} Geom&Factor_{CSA-CSA} =\\ =\Bigl \langle \Big (&(\sigma ^{E}_{xx}-\sigma ^{E}_{zz}) \cdot (\sigma ^{F}_{xx}-\sigma ^{F}_{zz}) \cdot P_2(cos\measuredangle (\vec {X}^{E},\vec {X}^{F}))+\\&+(\sigma ^{E}_{xx}-\sigma ^{E}_{zz}) \cdot (\sigma ^{F}_{yy}-\sigma ^{F}_{zz}) \cdot P_2(cos\measuredangle (\vec {X}^{E},\vec {Y}^{F}))+\\&+(\sigma ^{E}_{yy}-\sigma ^{E}_{zz}) \cdot (\sigma ^{F}_{xx}-\sigma ^{F}_{zz}) \cdot P_2(cos\measuredangle (\vec {Y}^{E},\vec {X}^{F}))+\\&+(\sigma ^{E}_{yy}-\sigma ^{E}_{zz}) \cdot (\sigma ^{F}_{yy}-\sigma ^{F}_{zz}) \cdot P_2(cos\measuredangle (\vec {Y}^{E},\vec {Y}^{F}))\Big ) \Bigr \rangle \end{aligned} \end{aligned}$$

where $r_{ij}$ is the length of the vector $\vec {r}_{ij}$ between *i* and *j* nuclei, $\sigma ^i_{k}$ is the principal value of the CSA tensor ($k= xx,yy,zz$) for nucleus *i*, and $\vec {X}^{i}$ and $\vec {Y}^{i}$ are the vectors along X and Y respectively, which are the principal axes of the nucleus *i* CSA tensor.

As mentioned before, the geometrical factor is directly related to the protein dihedral angles: $GeomFactor=GeomFactor(\phi , \psi )$, so the CCR rates can be the source of protein structural information. It should be emphasized, however, that the structural contribution is not readily separable from the dynamical one and therefore, we need to make assumptions about the dynamical contribution. In the case of folded proteins, overall rotational tumbling time is typically a sufficiently accurate representation of local dynamics. Nonetheless, even folded proteins can exhibit certain dynamical diversity, for instance, terminal fragments of the chain are often more mobile than the fragments involved in secondary and tertiary structural elements. The diversity of dynamics is even more pronounced in the case of intrinsically disordered proteins (IDPs), which are highly mobile and lack stable tertiary structures. In this case, local dynamics dominates over the overall tumbling of the molecule, which leads to substantially different dynamics for each residue. Thus, the dynamics has to be estimated separately for each residue (Ceccolini et al. 2024).

Another difficulty related to protein structural information is the non-injective character of the geometrical functions: typically, a multitude of different ($\phi$, $\psi$) distributions can lead to the same CCR rate. As the shape of each of the CCR rates’ geometrical dependencies is different, using several rates can resolve this ambiguity (Kloiber and Konrat 2000b). This approach, however, does not take into account that a single residue may transiently adopt different structures. Even folded proteins exhibit certain mobility and structural diversity. Yet, while the dihedral angles of most residues of a folded proteins vary only to a very limited extent, the residues of IDPs have to be described with a wide distribution of dihedral angles, which reflects the more diverse structural ensemble. In the case of very dispersed conformational distribution, the mathematical problem of back-calculation of the ensemble using the averaged observables is under-determined and requires additional assumptions in order to be solved, even when using several different CCR rates. One possible approach is to use the maximum entropy method (Kauffmann et al. 2021). Still, to increase the accuracy of the result, we need to measure many CCR rates, preferably with diverse geometrical dependencies.

In the past, many CCR rates have been exploited: ${\textrm{H}^\textrm{N}_{(\textrm{i})}}{\textrm{N}_{(\textrm{i})}}$ DD–${\textrm{H}^\alpha _{({i-1})}}{\textrm{C}^\alpha _{({i-1})}}$ DD (Reif et al. 1997; Yang and Kay 1998; Chiarparin et al. 1999; Pelupessy et al. 1999a), ${\textrm{H}^\textrm{N}_{(\textrm{i})}}{\textrm{N}_{(\textrm{i})}}$ DD–${\textrm{H}^\alpha _{(\textrm{i})}}{\textrm{C}^\alpha _{(\textrm{i})}}$ DD (Pelupessy et al. 1999b), ${\textrm{H}^\alpha _{({i-1})}}{\textrm{C}^\alpha _{({i-1})}}$ DD–$\textrm{C}'_{( i-1)}$ CSA (Yang et al. 1997, 1998; Chiarparin et al. 1999), ${\textrm{H}^\alpha _{(\textrm{i})}}{\textrm{C}^\alpha _{(\textrm{i})}}$ DD–$\textrm{C}'_{( i-1)}$ CSA (Kloiber and Konrat 2000a),${\textrm{H}^\textrm{N}_{(\textrm{i})}}{\textrm{N}_{(\textrm{i})}}$ DD–$\textrm{C}'_{(\textrm{i})}$ CSA (Kloiber and Konrat 2000b), ${\textrm{H}^\textrm{N}_{(\textrm{i})}}{\textrm{N}_{(\textrm{i})}}$ DD–${\textrm{N}_{({ i}-1)}}$ CSA (Tjandra et al. 1996), ${\textrm{H}^\alpha _{({i-1})}}{\textrm{C}^\alpha _{({i-1})}}$ DD– ${\textrm{H}^\alpha _{(\textrm{i})}}{\textrm{C}^\alpha _{(\textrm{i})}}$ DD (Chiarparin et al. 2000), $\textrm{C}'_{(\textrm{i})}$ CSA–$\textrm{C}'_{( i-1)}$ CSA (Skrynnikov et al. 2000). However, all of these experiments were carried out on folded proteins with relatively rigid structures, whereas our goal is to apply the CCR approach to more dynamic systems. In particular, in the future we would like to apply them to IDPs.

Most of the CCR experiments published to date are not suitable for IDPs as their dimensionality is too low: In two-dimensional (2D) or three-dimensional (3D) spectra, the level of peak overlap is very high. This is suboptimal, because in CCR experiments information is obtained from peak heights. To overcome this problem we need to increase the dimensionality of the experiment (Grudziąż et al. 2018). To date, only two CCR experiments have been published in a 4D version (Stanek et al. 2013; Kauffmann et al. 2020). Another reason why many previous experiments are unsuitable for IDPs is that they use the so-called ‘J-resolved’ approach, where the CCR rates are measured by comparing the heights (or linewidths) of multiplet components. As each peak is split, the problem of peak overlap is even more pronounced. For IDPs, it is beneficial to measure the spectrum twice—in two different versions: the transfer version and the reference version. In the transfer version only the magnetization obtained with the CCR-mediated transfer of coherence is selected; in the reference version the selected magnetization is obtained without the CCR-mediated transfer. The CCR rates are calculated using the corresponding peak heights in both versions of the experiment (Brutscher 2000):

12 $$\begin{aligned} \varGamma = \frac{1}{Tc}\cdot \mathrm{{{\,\textrm{artanh}\,}}}{\frac{I_{trans}}{I_{ref}}} \end{aligned}$$

where *Tc* is the time of CCR evolution and $I_{trans}$ and $I_{ref}$ are the peak heights in transfer and reference versions. The transfer version of the experiment is typically much less sensitive, so it is advisable to carry out more scans than in the reference version. This should be taken into account in Eq. 12: The $\frac{I_{trans}}{I_{ref}}$ ratio should be multiplied by $\frac{NS_{ref}}{NS_{trans}}$, where *NS* denotes the number of scans. Such an approach was first used by Tjandra et al. (1996), and it is sometimes referred to as a ‘coherence transfer experiment’ (Brutscher 2000) or a ‘quantitative gamma approach’ (Carlomagno and Griesinger 2000). We use the latter term in this study.

In this paper we present a complete set of eight CCR experiments tailored for proteins. All experiments are four-dimensional (4D), which reduces the problem of spectral crowding (Grudziąż et al. 2018). Each experiment yields a different rate, with different geometrical factors (some depending on $\phi$, some on $\psi$, and some on both). All experiments use the quantitative gamma approach. When combined with the maximum entropy approach (Kauffmann et al. 2021), this set of experiments allows for a complete analysis of the dihedral angles distribution for each protein residue. While in this work the experiments are applied to the mostly structured protein Ubiquitin to validate them against data from orthogonal NMR methods, they are designed to investigate IDPs in future studies.

## Methods

### NMR experiments

**Fig. 1:**
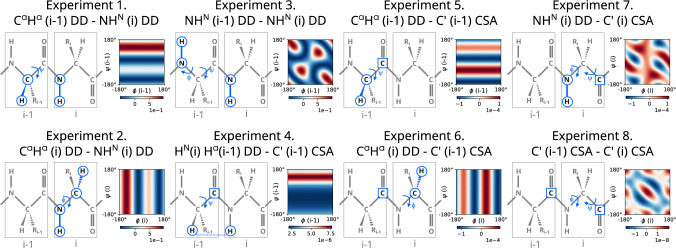
Scheme showing the nuclei and dihedral angles involved in the CCR effect for each experiment. For each CCR rate the map of the geometrical contribution (dependence on the dihedral angles $\phi$—shown on a horizontal axis, and $\psi$—shown on a vertical axis) is shown. Indices *i* and $i-1$ indicate the relative positions of residues involved, in each case the signal detection was performed on the $i^{th}$ amide proton

We developed a set of eight experiments, each providing a different CCR rate. In each case, only one CCR rate at a time is measured; spin evolution due to all other substantial CCR mechanisms is either refocused or leads to coherence that is not converted to observable magnetization. All experiments are four-dimensional. The directly detected nucleus is amide proton and the indirectly detected ones are amide nitrogen, carbonyl carbon, and alpha carbon. Carbon nuclei belong to the same amino acid residue as the amide group, or the preceding one, depending on the experiment. Information on measured nuclei, CCR rates, and the angles that we can extract from each rate is summarized in Table 1 and Fig. 1.

**Table 1:** Information on peak types, CCR rates, and angles

Experiment	CCR rate	Peak type	Angles
1	$\textrm{C}^{\alpha }_{(i-1)}\textrm{H}^{\alpha }_{(i-1)}$ DD–$\textrm{N}_{(i)}\textrm{H}^{\textrm{N}}_{(i)}$ DD	$\textrm{H}^{\textrm{N}}_{(\textrm{i})}-\textrm{N}_{(\textrm{i})}$ – $\textrm{C}'_{( i-1)}$ – $\textrm{C}^\alpha _{( i-1)}$	${\psi _{(i-1)}}$
2	$\textrm{C}^{\alpha }_{(i)}\textrm{H}^{\alpha }_{(i)}$ DD–$\textrm{N}_{(i)}\textrm{H}^{\textrm{N}}_{(i)}$ DD	$\textrm{H}^{\textrm{N}}_{(\textrm{i})}-\textrm{N}_{(\textrm{i})}$ – $\textrm{C}'_{( i-1)}$ – $\textrm{C}^\alpha _{(\textrm{i})}$	${\phi _{(\textrm{i})}}$
3	$\textrm{N}_{(i-1)}\textrm{H}^{\textrm{N}}_{(i-1)}$ DD–$\textrm{N}_{(i)}\textrm{H}^{\textrm{N}}_{(i)}$ DD	$\textrm{H}^{\textrm{N}}_{(\textrm{i})}-\textrm{N}_{(\textrm{i})}$ – $\textrm{C}'_{( i-1)}$ – $\textrm{C}^\alpha _{( i-1)}$	${\psi _{(i-1)},\phi _{(i-1)}}$
4	$\textrm{H}^{\textrm{N}}_{(i)}\textrm{H}^{\alpha }_{(i-1)}$ DD–$\textrm{C}'_{(i-1)}$ CSA	$\textrm{H}^{\textrm{N}}_{(\textrm{i})}-\textrm{N}_{(\textrm{i})}$ – $\textrm{C}'_{( i-1)}$ – $\textrm{C}^\alpha _{( i-1)}$	${\psi _{(i-1)}}$
5	$\textrm{C}^{\alpha }_{(i-1)}\textrm{H}^{\alpha }_{(i-1)}$ DD–$\textrm{C}'_{(i-1)}$ CSA	$\textrm{H}^{\textrm{N}}_{(\textrm{i})}-\textrm{N}_{(\textrm{i})}$ – $\textrm{C}'_{( i-1)}$ – $\textrm{C}^\alpha _{( i-1)}$	${\psi _{(i-1)}}$
6	$\textrm{C}^{\alpha }_{(i)}\textrm{H}^{\alpha }_{(i)}$ DD–$\textrm{C}'_{(i-1)}$ CSA	$\textrm{H}^{\textrm{N}}_{(\textrm{i})}-\textrm{N}_{(\textrm{i})}$ – $\textrm{C}'_{( i-1)}$ – $\textrm{C}^\alpha _{(\textrm{i})}$	${\phi _{(\textrm{i})}}$
7	$\textrm{N}_{(i)}\textrm{H}^{\textrm{N}}_{(i)}$ DD–$\textrm{C}'_{(i)}$ CSA	$\textrm{H}^{\textrm{N}}_{(\textrm{i})}-\textrm{N}_{(\textrm{i})}$ – $\textrm{C}'_{(\textrm{i})}$ – $\textrm{C}^\alpha _{(\textrm{i})}$	${\psi _{(\textrm{i})},\phi _{(\textrm{i})}}$
8	$\textrm{C}'_{(i-1)}$ CSA–$\textrm{C}'_{(i)}$ CSA	$\textrm{H}^{\textrm{N}}_{(\textrm{i})}-\textrm{N}_{(\textrm{i})}$ – $\textrm{C}'_{( i-1)}$ – $\textrm{C}^\alpha _{(\textrm{i})}$	${\psi _{(\textrm{i})},\phi _{(\textrm{i})}}$

Indices *i* and $i-1$ indicate the relative positions of the residues involved

In all the experiments we conducted, we used the quantitative gamma approach (Tjandra et al. 1996; Brutscher 2000), which required two versions of the experiment to be carried out: a reference version and a transfer version. We designed each pulse sequence in the same way. After the relaxation delay d1, the equilibrium $^{13}$C and $^{15}$N polarization was eliminated, using for each of these channels a pair of 90 degree pulses, each followed by a gradient. Then, four main steps were taken, namely:

1. Create the coherence needed for CCR evolution
2. CCR block: carry out the CCR evolution. At the end of it the coherence consists of two parts: ‘auto’ (identical to the initial state at the beginning of the CCR block) and ‘cross’ (originating from the CCR evolution of the original state). In the reference and transfer versions the phases of the last pulses of the block are adjusted to select the desired component: ‘auto’ and ‘cross’, respectively.
3. Unifying block (present only if the forms of ‘auto’ and ‘cross’ coherences after the CCR block are different): convert the coherence to the same state (only differing in terms of intensity) for both versions of the experiment, reference and transfer. In the reference version this block should keep the coherence unchanged, while in the transfer version it should refocus anti-phase magnetization with respect to certain nuclei. Both versions should be as similar as possible regarding the delays and the number of pulses, to provide quantitative results of the CCR rates.
4. Transfer the coherence to the observable magnetization and acquisition. A sensitivity enhancement block with gradient coherence selection (Kay et al. 1992; Schleucher et al. 1993) was used in each experiment, therefore the $\textrm{H}^\textrm{N}_{x,y(i)}$operator, used in the following subsections in the schemes of coherence transfer pathway description, stands for phase-modulated signal of the following form: ${{\textrm{H}^\textrm{N}_{x(i)}}\sin (\varOmega _\textrm{N} \,\textrm{t}_3)\pm {\textrm{H}^\textrm{N}_{y(i)}}\cos (\varOmega _\textrm{N} \,\textrm{t}_3)}$ (where $\varOmega _N$ is the nitrogen frequency and $t_3$ is the nitrogen evolution time).

More detailed descriptions of all experiments are given in the subsections below.

#### Experiment 1. $\textrm{C}^{\alpha }_{(i-1)}\textrm{H}^{\alpha }_{(i-1)}$ DD–$\textrm{N}_{(i)}\textrm{H}^{\textrm{N}}_{(i)}$ DD

**Fig. 2:**
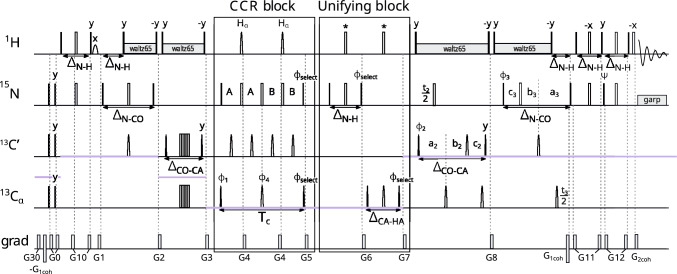
Pulse sequence of the experiment 1 ($\textrm{C}^{\alpha }_{(i-1)}\textrm{H}^{\alpha }_{(i-1)}$ DD–$\textrm{N}_{(i)}\textrm{H}^{\textrm{N}}_{(i)}$ DD CCR). The evolution of ${\textrm{C}^{\alpha }}$ is in constant-time mode during CCR evolution: $A = (\textrm{T}_{c}+t_{1})/4$ and $B = (\textrm{T}_{c}-t_{1})/4$, C$'$ evolution is in (semi)constant-time mode (Grzesiek and Bax 1993): $a_2=(\varDelta _{\mathrm{CO-CA}}+t_2)/2, \ b_2=t_2(1-\varDelta _{\mathrm{CO-CA}})/(2t^{max}_{2}), \ c_2=\varDelta _{\mathrm{CO-CA}}(1-t_2)/(2t^{max}_{2})$ and N evolution is in (semi)constant-time mode: $a_3=(\varDelta _{\mathrm{N-CO}}+t_3)/2, \ b_3=t_3(1-\varDelta _{\mathrm{N-CO}})/(2t^{max}_{3}), \ c_3=\varDelta _{\mathrm{N-CO}}(1-t_3)/(2t^{max}_{3})$. The semi-constant or constant time mode is chosen automatically, based on the number of sampling points and spectral width in a given dimension. The delays were set as follows: $\varDelta _{\mathrm{N-H}}$=5.4 ms, $\varDelta _{\mathrm{N-CO}}$=31 ms, $\varDelta _{\mathrm{CO-N}}$=29 ms, $\varDelta _{\mathrm{CO-CA}}$=9.1 ms, and $\varDelta _{\mathrm{CA-HA}}$=3.4 ms. Total CCR rate evolution time is set as $\textrm{T}_{c}$=28.6 ms. Unless noted explicitly, pulse phases are set to x. Phase $\phi _{select}$ depends on the version of the experiment: for reference experiment x, for transfer experiment y. The value of individual phase cycles are $\phi _{1}=x,-x$, $\phi _{2}=2(x),2(-x)$, $\phi _{3}=x$, $\phi _{4}=4(x),4(y),4(-x),4(-y)$, $\psi =y$ and receiver $\phi _{r}=\phi _{1}+\phi _{2}+2\cdot \phi _{4}$. Quadrature detection is achieved by States-TPPI (Marion et al. 1989) for ${\textrm{C}^{\alpha }}$ (change of $\phi _{1}$ phase) and C$'$ (change of $\phi _{2}$ phase) evolutions, and echo-antiecho (Kay et al. 1992; Schleucher et al. 1993) for N evolution (change of $\phi _{3}$ and $\psi$ phases and change of $G_{1coh}$ amplitude sign). Selective pulses for C$'$ and ${\textrm{C}^{\alpha }}$ spins were Q3 (for 180$^\circ$ pulses) and Q5 (for 90$^\circ$ pulses) (Emsley and Bodenhausen 1992). The selective 180$^\circ$ pulse on proton channel (marked ${\textrm{H}_{\alpha }}$) affects only ${\textrm{H}^{\alpha }}$ and was prepared by Bruker WaveMaker tool, using offset=4.3 ppm, bandwidth=3 ppm, reburp shape (Geen and Freeman 1991). Simultaneous inversion of ${\textrm{C}^{\alpha }}$ and C$'$ spins was achieved using 6-element composite pulse (Shaka 1985). The pulses labelled by a star ("*") were executed only in the transfer version of the experiment. Rectangles represent hard pulses while rounded cones symbolize shaped pulses; the empty ones are 180$^\circ$ pulses and the filled ones are 90$^\circ$ pulses. Gradients with numbers from 1 to 12 are cleaning gradients. Gradients $G_{1coh}$ and $G_{2coh}$ are used for coherence selection in echo-antiecho quadrature detection. Proton decoupling is performed with composite pulse scheme waltz65 (Zhou et al. 2007) and nitrogen decoupling during acquisition is performed with composite pulse scheme garp (Shaka et al. 1985). The CCR and unifying blocks are indicated by rectangle boxes

The first experiment measures the CCR effect between $\textrm{C}^{\alpha }_{(i-1)}{\textrm{H}^{\alpha }_{(i-1)}}$ DD and $\textrm{N}_{({ i})}\textrm{H}^{\textrm{N}}_{({ i})}$ DD. It yields information on the ${\psi }$ angle (peak ${\textrm{H}^{\textrm{N}}_{(\textrm{i})}-\textrm{N}_{(\textrm{i})}}$ – $\textrm{C}'_{( i-1)}$ – $\textrm{C}^\alpha _{( i-1)}$informs on ${\psi _{(i-1)}}$). Our pulse sequence is shown in Fig. 2 and is based on the standard 4D HNCOCA pulse sequence (Bax and Ikura 1991; Yang and Kay 1999). The coherence transfer before the CCR block is as follows:

$$\begin{aligned} & \textrm{H}^\textrm{N}_{z(i)}\xrightarrow []{J_{NH}} 2\textrm{H}^\textrm{N}_{z(i)}\textrm{N}_\mathrm{{z(i)}}\xrightarrow [{J_{NC'}}]{J_{NH}} 2\textrm{C}'_{z(i-1)}\textrm{N}_\mathrm{{z(i)}}\xrightarrow []{J_{C'C^{\alpha }}} \\ & \xrightarrow []{J_{C'C^{\alpha }}} 4\textrm{C}^{\alpha }_{z(i-1)}\textrm{C}'_{z(i-1)}\textrm{N}_\mathrm{{z(i)}}\Rightarrow \text {CCR block} \end{aligned}$$

**Fig. 3:**
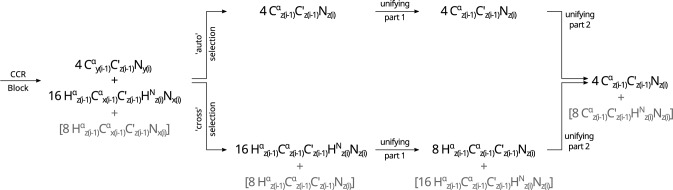
Scheme showing the difference in coherence transfer between transfer and reference version of the Experiment 1( $\textrm{C}^{\alpha }_{(i-1)}\textrm{H}^{\alpha }_{(i-1)}$ DD–$\textrm{N}_{(i)}\textrm{H}^{\textrm{N}}_{(i)}$ DD)

The CCR block contains two types of evolution: CCR evolution, and constant-time chemical shift evolution of $\textrm{C}^\alpha _{( i-1)}$. Two substantial unwanted CCR evolutions, i.e. $\textrm{N}_{(i)}\textrm{H}^\textrm{N}_{(i)}$ DD–$\textrm{C}^{\alpha }_{(i-1)}$ CSA and $\textrm{C}^{\alpha }_{(i-1)}$ CSA–$\textrm{N}_{(i)}$ CSA were refocused during the CCR block by the first and third 180$^\circ$ pulses acting on nitrogen nuclei. Another unwanted CCR evolution, i.e. $\textrm{C}^{\alpha }_{(i-1)}\textrm{H}^{\alpha }_{(i-1)}$ DD–$\textrm{N}_{(i)}$ CSA, did occur during the CCR block, but the resulting coherences (on the schemes of coherence transfer below shown in squared brackets) were not transferred into an observable magnetization. To minimize the loss of coherence due to ${\textrm{C}^{\alpha }-\textrm{C}^{\beta }}$ J-coupling evolution, we set the overall evolution time to the inverse of the ${\textrm{C}^{\alpha }-\textrm{C}^{\beta }}$ J-coupling constant, that is, 28.6 ms. During the CCR block, the initial 4$\textrm{C}^{\alpha }_{y(i-1)}$ $\textrm{C}'_{z(i-1)}$ $\textrm{N}_{y(i)}$ coherence is partially converted into 16$\textrm{H}^{\alpha }_{z(i-1)}$ $\textrm{C}^{\alpha }_{x(i-1)}$ $\textrm{C}'_{z(i-1)}$ $\textrm{N}_{x(i)}$ $\textrm{H}^\textrm{N}_{z(i)}$ coherence, so we can use different phases of the last 90$^\circ$ pulses (acting on $\textrm{C}^{\alpha }$ and N) to select each component: The x-pulses allow us to observe the unchanged coherence, while the y-pulses allow us to observe the component originating from CCR evolution. In the undesired coherence the transverse component remains, thus it is dephased by the following gradient (G5). During the unifying block, the 16$\textrm{H}^{\alpha }_{z(i-1)}$ $\textrm{C}^{\alpha }_{z(i-1)}$ $\textrm{C}'_{z(i-1)}$ $\textrm{N}_\mathrm{{z(i)}}$ $\textrm{H}^\textrm{N}_{z(i)}$ five-spin order is transferred back to a 4$\textrm{C}^{\alpha }_{z(i-1)}$ $\textrm{C}'_{z(i-1)}$ $\textrm{N}_\mathrm{{z(i)}}$ three-spin order. This is achieved by the evolution of the J$_{\textrm{NH}}$ coupling and the consecutive evolution of the J$_{\textrm{C}^{\alpha }\textrm{H}^{\alpha }}$ coupling; see Fig. 3.

Next, during the transfer of the coherence to the observable magnetization, we allow for the evolution of the chemical shifts of $\textrm{C}'_{( i-1)}$ and $\textrm{N}_{(i)}$. The coherence transfer after the CCR and unifying blocks is as follows:

$$\begin{aligned} &4\textrm{C}^{\alpha }_{z(i-1)}\textrm{C}'_{z(i-1)}\textrm{N}_\mathrm{{z(i)}}+ [8\textrm{C}^{\alpha }_{z(i-1)}\textrm{C}'_{z(i-1)}\textrm{H}^\textrm{N}_{z(i)}\textrm{N}_\mathrm{{z(i)}}] \xrightarrow []{J_{C'C^{\alpha }}} \\&\quad \xrightarrow []{J_{C'C^{\alpha }}} 2\textrm{C}'_{z(i-1)}\textrm{N}_\mathrm{{z(i)}}+ [4\textrm{C}'_{z(i-1)}\textrm{H}^\textrm{N}_{z(i)}\textrm{N}_\mathrm{{z(i)}}]\xrightarrow [{J_{NC'}}]{J_{NH}}\\&\quad \xrightarrow [{J_{NC'}}]{J_{NH}} 2\textrm{H}^\textrm{N}_{z(i)}\textrm{N}_\mathrm{{z(i)}}+ [\textrm{N}_\mathrm{{z(i)}}] \xrightarrow []{J_{HN}} \\&\quad \xrightarrow []{J_{HN}} \textrm{H}^\textrm{N}_{x,y(i)}+ [\mathrm{multiple-quantum \quad coherence}] \end{aligned}$$

Previously, this rate was measured by Reif et al. (1997) and by Yang and Kay (1998) as a 3D HN(CO)CA J-resolved experiment, and by Pelupessy et al. (1999a) in a 2D quantitative gamma version (without carbon evolution). The latter solution is similar to ours (a quantitative gamma approach with the same operator at the start of the CCR block), but there are some important differences. In the 2D version, the lack of $\textrm{C}^{\alpha }$ evolution made it possible to retain the initial coherence at the end of the CCR block in the transfer version too, as the proper shifting of the $\textrm{H}^{\alpha }$ pulses led to $\textrm{C}^{\alpha }$- $\textrm{H}^{\alpha }$ J-coupling evolution. In a 3D version, this would require real-time $\textrm{C}^{\alpha }$ evolution (after the CCR block), which would, in our opinion, not be optimal due to the occurrence of J$_{\textrm{C}^{\alpha }\textrm{C}^{\beta }}$ modulation. We therefore included C$^{\alpha }$ chemical shift evolution in the CCR block and added a unifying block directly after it.

#### Experiment 2. $\textrm{C}^{\alpha }_{(i)}\textrm{H}^{\alpha }_{(i)}$ DD–$\textrm{N}_{(i)}\textrm{H}^{\textrm{N}}_{(i)}$ DD

**Fig. 4:**
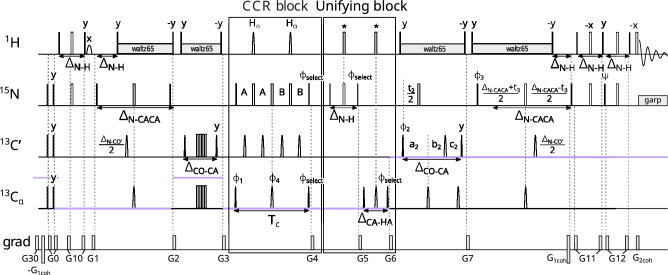
Pulse sequence of the experiment 2 ($\textrm{C}^{\alpha }_{(i)}\textrm{H}^{\alpha }_{(i)}$ DD–$\textrm{N}_{(i)}\textrm{H}^{\textrm{N}}_{(i)}$ DD CCR). The evolution of ${\textrm{C}^{\alpha }}$ is in constant-time mode during CCR evolution: $A = (\textrm{T}_{c}+t_{1})/4$ and $B = (\textrm{T}_{c}-t_{1})/4$, C$'$ evolution is in (semi)constant-time mode: $a_2=(\varDelta _{\mathrm{N-CO}}+t_2)/2, \ b_2=t_2(1-\varDelta _{\mathrm{N-CO}})/(2t^{max}_{2}), \ c_2=\varDelta _{\mathrm{N-CO}}(1-t_2)/(2t^{max}_{2})$ and N evolution is in constant-time mode. The semi-constant (Grzesiek and Bax 1993) or constant time mode is chosen automatically, based on the number of sampling points and spectral width in a given dimension. The delays were set as follows: $\varDelta _{\mathrm{N-H}}$ = 5.4 ms, $\varDelta _{\mathrm{N-CO'}}$ = 33.5 ms, $\varDelta _{\mathrm{N-CACA}}$ = 50 ms, $\varDelta _{\mathrm{CO-CA}}$ = 9.1 ms, and $\varDelta _{\mathrm{CA-HA}}$ = 3.4 ms. Total CCR rate evolution time is set as $\textrm{T}_{c}$ = 28.6 ms. Unless noted explicitly, pulse phases are set to x. Phase $\phi _{select}$ depends on the version of the experiment: for reference experiment x, for transfer experiment y. The value of individual phase cycles are $\phi _{1}=x,-x$, $\phi _{2}=2(x),2(-x)$, $\phi _{3}=x$, $\phi _{4}=4(x),4(y),4(-x),4(-y)$, $\psi =y$ and receiver $\phi _{r}=\phi _{1}+\phi _{2}+2\cdot \phi _{4}$. Quadrature detection is achieved by States-TPPI (Marion et al. 1989) for ${\textrm{C}^{\alpha }}$ (change of $\phi _{1}$ phase) and C$'$ (change of $\phi _{2}$ phase) evolutions, and echo-antiecho (Kay et al. 1992; Schleucher et al. 1993) for N evolution (change of $\phi _{3}$ and $\psi$ phases and change of $G_{1coh}$ amplitude sign). Selective pulses for C$'$ and ${\textrm{C}^{\alpha }}$ spins were Q3 (for 180$^\circ$ pulses) and Q5 (for 90$^\circ$ pulses) (Emsley and Bodenhausen 1992) The selective 180$^\circ$ pulse on proton channel (marked ${\textrm{H}_{\alpha }}$) affects only ${\textrm{H}^{\alpha }}$ and was prepared by Bruker WaveMaker tool, using offset = 4.3 ppm, bandwidth = 3 ppm, reburp shape (Geen and Freeman 1991). Simultaneous inversion of ${\textrm{C}^{\alpha }}$ and C$'$ spins was achieved using 6-element composite pulse (Shaka 1985). The pulses labelled by a star (”*”) were executed only in the transfer version of the experiment. Rectangles represent hard pulses while rounded cones symbolize shaped pulses; the empty ones are 180$^\circ$ pulses and the filled ones are 90$^\circ$ pulses. Gradients with numbers from 1 to 12 are cleaning gradients. Gradients $G_{1coh}$ and $G_{2coh}$ are used for coherence selection in echo-antiecho quadrature detection. Proton decoupling is performed with composite pulse scheme waltz65 (Zhou et al. 2007) and nitrogen decoupling during acquisition is performed with composite pulse scheme garp (Shaka et al. 1985). The CCR and unifying blocks are indicated by rectangle boxes

The second experiment measures the CCR effect between $\textrm{C}^{\alpha }_{(i)}\textrm{H}^{\alpha }_{(i)}$ DD and $\textrm{N}_{(i)}\textrm{H}^{\textrm{N}}_{(i)}$ DD. From the peak ${\textrm{H}^{\textrm{N}}_{(\textrm{i})}-\textrm{N}_{(\textrm{i})}}$ – $\textrm{C}'_{( i-1)}$ – $\textrm{C}^\alpha _{(\textrm{i})}$we get information on the ${\phi _{(\textrm{i})}}$ angle. The first measurement of this rate was proposed by Pelupessy et al. (1999b), whose 3D HNCA J-resolved experiment made it possible to simultaneously measure $\textrm{C}^{\alpha }_{(i-1)}\textrm{H}^{\alpha }_{(i-1)}$ DD–$\textrm{N}_{(i)}\textrm{H}^{\textrm{N}}_{(i)}$ DD and $\textrm{C}^{\alpha }_{(i)}\textrm{H}^{\alpha }_{(i)}$ DD–$\textrm{N}_{(i)}\textrm{H}^{\textrm{N}}_{(i)}$ DD rates. In our experiment, the coherence transfer pathway is based on a 4D HNCO$_{(i-1)}$CA$_{(i)}$ experiment (Konrat et al. 1999), so it yields a single peak per residue, which provides better peak separation and also makes it possible to measure only the $\textrm{C}^{\alpha }_{(i)}\textrm{H}^{\alpha }_{(i)}$ DD–$\textrm{N}_{(i)}\textrm{H}^{\textrm{N}}_{(i)}$ DD rate. The pulse sequence is shown in Fig. 4. In this experiment, the coherence transfer starts as follows:

$$\begin{aligned} &\textrm{H}^\textrm{N}_{z(i)}\xrightarrow []{J_{NH}} 2\textrm{H}^\textrm{N}_{z(i)}\textrm{N}_\mathrm{{z(i)}}\xrightarrow [J_{NH},J_{NC'}]{^1J_{NC^{\alpha }},^2J_{NC^{\alpha }}} 8\textrm{C}^{\alpha }_{z(i-1)}\textrm{C}'_{z(i-1)}\textrm{N}_\mathrm{{z(i)}}\textrm{C}^{\alpha }_{z(i)}\quad\xrightarrow []{J_{C'C^{\alpha }}} \\ &\xrightarrow []{J_{C'C^{\alpha }}} 4\textrm{C}'_{z(i-1)}\textrm{N}_\mathrm{{z(i)}}\textrm{C}^{\alpha }_{z(i)}\Rightarrow \text {CCR block} \end{aligned}$$

The CCR block and the unifying block are identical as in Experiment 1 ($\textrm{C}^{\alpha }_{(i-1)}\textrm{H}^{\alpha }_{(i-1)}$ DD–$\textrm{N}_{(i)}\textrm{H}^{\textrm{N}}_{(i)}$ DD). Analogously, the $\textrm{N}_{(i)}\textrm{H}^\textrm{N}_{(i)}$ DD–$\textrm{C}^{\alpha }_{(i)}$ CSA and $\textrm{N}_{(i)}$ CSA–$\textrm{C}^{\alpha }_{(i)}$ CSA were refocused during the CCR block, and $\textrm{C}^{\alpha }_{(i)}\textrm{H}^{\alpha }_{(i)}$ DD–$\textrm{N}_{(i)}$ CSA was not refocused, but did not lead to an observable magnetization (on the coherence transfer schemes below it is shown in squared brackets).

In the final fragment of the pulse sequence, we allow for the evolution of the chemical shifts of $\textrm{C}'_{( i-1)}$and $\textrm{N}_{(i)}$. The evolution of the $\textrm{N}_{(i)}$ chemical shift is executed in constant-time mode because the INEPT delay is very long (50 ms) and there is no need to implement a semi-constant time evolution option. The coherence transfer of this fragment of the pulse sequence is as follows:

$$\begin{aligned}&4\textrm{C}'_{z(i-1)}\textrm{N}_\mathrm{{z(i)}}\textrm{C}^{\alpha }_{z(i)}+ [8\textrm{C}^{\alpha }_{z(i-1)}\textrm{C}'_{z(i-1)}\textrm{H}^\textrm{N}_{z(i)}\textrm{N}_\mathrm{{z(i)}}] \xrightarrow []{J_{C'C^{\alpha }}} \\&\quad \xrightarrow []{J_{C'C^{\alpha }}} 8\textrm{C}^{\alpha }_{z(i-1)}\textrm{C}'_{z(i-1)}\textrm{N}_\mathrm{{z(i)}}\textrm{C}^{\alpha }_{z(i)}+ [4\textrm{C}'_{z(i-1)}\textrm{H}^\textrm{N}_{z(i)}\textrm{N}_\mathrm{{z(i)}}] \xrightarrow [J_{NH},J_{NC'}]{^1J_{NC^{\alpha }},^2J_{NC^{\alpha }}} \\&\quad \xrightarrow [J_{NH},J_{NC'}]{^1J_{NC^{\alpha }},^2J_{NC^{\alpha }}} 2\textrm{H}^\textrm{N}_{z(i)}\textrm{N}_\mathrm{{z(i)}}+ [4\textrm{C}^{\alpha }_{z(i-1)}\textrm{N}_\mathrm{{z(i)}}\textrm{C}^{\alpha }_{z(i)}] \xrightarrow []{J_{NH}} \\&\quad \xrightarrow []{J_{HN}} \textrm{H}^\textrm{N}_{x,y(i)}+ [\mathrm{multiple-quantum\quad coherence}] \end{aligned}$$

#### Experiment 3. $\textrm{N}_{(i-1)}\textrm{H}^{\textrm{N}}_{(i-1)}$ DD–$\textrm{N}_{(i)}\textrm{H}^{\textrm{N}}_{(i)}$ DD

**Fig. 5:**
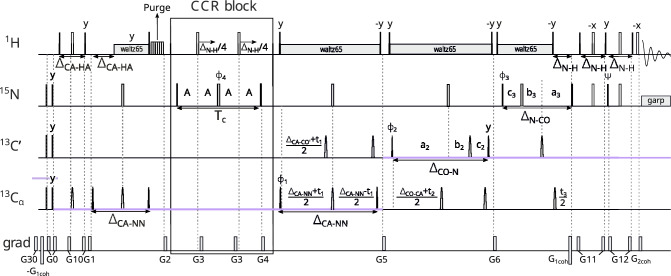
Pulse sequence of the experiment 3 ($\textrm{N}_{(i-1)}\textrm{H}^{\textrm{N}}_{(i-1)}$ DD–$\textrm{N}_{(i)}\textrm{H}^{\textrm{N}}_{(i)}$ DD CCR). The evolution of ${\textrm{C}^{\alpha }}$ is in constant-time mode, C$'$ evolution is in (semi)constant-time mode: $a_2=(\varDelta _{\mathrm{CO-N}}+t_2)/2, \ b_2=t_2(1-\varDelta _{\mathrm{CO-N}})/(2t^{max}_{2}), \ c_2=\varDelta _{\mathrm{CO-N}}(1-t_2)/(2t^{max}_{2})$ and N evolution is in (semi)constant-time mode: $a_3=(\varDelta _{\mathrm{N-CO}}+t_3)/2, \ b_3=t_3(1-\varDelta _{\mathrm{N-CO}})/(2t^{max}_{3}), \ c_3=\varDelta _{\mathrm{N-CO}}(1-t_3)/(2t^{max}_{3})$. The semi-constant (Grzesiek and Bax 1993) or constant time mode is chosen automatically, based on the number of sampling points and spectral width in a given dimension. The delays were set as follows: $A = \textrm{T}_{c}/4$, $\textrm{T}_{c}$ = 28 ms, $\varDelta _{\mathrm{N-H}}$ = 5.4 ms, $\varDelta _{\mathrm{N-CO}}$ = 31 ms, $\varDelta _{\mathrm{CO-N}}$ = 29 ms, $\varDelta _{\mathrm{CA-NN}}$ = 55 ms, $\varDelta _{\mathrm{CO-CA}}$ = 9.1 ms, $\varDelta _{\mathrm{CA-CO'}}$ = 9.4 ms, and $\varDelta _{\mathrm{CA-HA}}$ = 3.4 ms. Unless noted explicitly, pulse phases are set to x. Phase $\phi _{select}$ depends on the version of the experiment: for reference experiment x, for transfer experiment y. The value of individual phase cycles are $\phi _{1}=x,-x$, $\phi _{2}=2(x),2(-x)$, $\phi _{3}=x$, $\phi _{4}=4(x),4(y),4(-x),4(-y)$, $\psi =y$ and receiver $\phi _{r}=\phi _{1}+\phi _{2}$. Quadrature detection is achieved by States-TPPI (Marion et al. 1989) for ${\textrm{C}^{\alpha }}$ (change of $\phi _{1}$ phase) and C$'$ (change of $\phi _{2}$ phase) evolutions, and echo-antiecho (Kay et al. 1992; Schleucher et al. 1993) for N evolution (change of $\phi _{3}$ and $\psi$ phases and change of $G_{2coh}$ amplitude sign). Selective pulses for C$'$ and ${\textrm{C}^{\alpha }}$ spins were Q3 (for 180$^\circ$ pulses) and Q5 (for 90$^\circ$ pulses) (Emsley and Bodenhausen 1992). The pulses labelled by arrow ("$\rightarrow$") were shifted by $\varDelta _{\mathrm{N-H}}$/4 to the right in the transfer version of the experiment. Rectangles represent hard pulses while rounded cones symbolize shaped pulses; the empty ones are 180$^\circ$ pulses and the filled ones are 90$^\circ$ pulses. Gradients with numbers from 1 to 12 are cleaning gradients. Gradients $G_{1coh}$ and $G_{2coh}$ are used for coherence selection in echo-antiecho quadrature detection. Proton decoupling is performed with composite pulse scheme waltz65 (Zhou et al. 2007) and nitrogen decoupling during acquisition is performed with composite pulse scheme garp (Shaka et al. 1985). The CCR and unifying blocks are indicated by rectangle boxes

The third experiment measures the CCR effect between $\textrm{N}_{(i-1)}\textrm{H}^{\textrm{N}}_{(i-1)}$ DD and $\textrm{N}_{(i)}\textrm{H}^{\textrm{N}}_{(i)}$ DD. From the peak ${\textrm{H}^{\textrm{N}}_{(\textrm{i})}}-\textrm{N}_{(\textrm{i})}$ – $\textrm{C}'_{( i-1)}$ – $\textrm{C}^\alpha _{( i-1)}$we get information on the $\psi _{(\mathrm{i-1})}$and $\phi _{({i-1})}$ angles simultaneously. This CCR rate was previously measured in two ways: Pelupessy et al. (2003b) proposed a 2D quantitative gamma experiment (only nitrogen and proton evolution), and later Vögeli (2017) proposed 3D experiments (HN(CA)CON and HNCA(CO)N), where a difference in relaxation between zero-quantum and double-quantum coherences was evaluated and each peak appeared as a multiplet (which is not optimal for IDPs). The coherence transfer pathway of our experiment differs substantially from those previously proposed (see Fig. 5), starting from the excitation of $\textrm{H}^{\alpha }$ nuclei instead of $\textrm{H}^\textrm{N}$ ones:

$$\begin{aligned} & \textrm{H}^{\alpha }_{z(i-1)}\xrightarrow []{J_{C^{\alpha }H^{\alpha }}} 2\textrm{H}^{\alpha }_{z(i-1)}\textrm{C}^{\alpha }_{z(i-1)}\xrightarrow [J_{C^{\alpha }H^{\alpha }}]{^{1}J_{C^{\alpha }N},^{2}J_{C^{\alpha }N}}\\ & \rightarrow 4\textrm{N}_{z(i-1)}\textrm{C}^{\alpha }_{z(i-1)}\textrm{N}_\mathrm{{z(i)}}\Rightarrow \text {CCR block} \end{aligned}$$

The CCR block is the same as in Pelupessy et al. (2003b).

The frequencies of the HA nuclei (which we excite at the beginning of the pulse sequence) are close to the frequency of water, therefore we add a purge pulse before the CCR block to crush the water magnetization.

During the CCR block, the CCR ($\textrm{N}_{(i-1)}\textrm{H}^{\textrm{N}}_{(i-1)}$ DD–$\textrm{N}_{(i)}\textrm{H}^{\textrm{N}}_{(i)}$ DD ) evolves, partially leading to the anti-phase magnetization of amide nitrogen nuclei with respect to amide protons. In the reference version of the experiment, the evolution of ${\textrm{J}_\textrm{NH}}$ coupling is refocused, thus the ‘auto’ component in this version of the experiment has the form of in-phase magnetization. In the transfer version, the two 180$^\circ$ proton pulses are shifted, leading to the evolution of ${\textrm{J}_{\textrm{NH}}}$ coupling. As a result, the ‘cross’ term in this version of the experiment has the form of in-phase magnetization (similarly to ‘auto’ component of the reference version). Therefore, no unifying block is employed in the pulse sequence. Three potentially significant unwanted CCR rates should be considered at this point. Evolution due to $\textrm{N}_{(i-1)}\textrm{H}^\textrm{N}_{(i-1)}$ DD–$\textrm{N}_{(i)}$ CSA and $\textrm{N}_{(i)}\textrm{H}^\textrm{N}_{(i)}$ DD–$\textrm{N}_{(i-1)}$ CSA was refocused in the CCR block by the 180$^\circ$ pulses acting on amide proton nuclei. However $\textrm{N}_{(i-1)}$ CSA–$\textrm{N}_{(i)}$ CSA did evolve, but the resulting coherence was not transferred to an observable one (on the coherence transfer scheme below it is shown in squared brackets).

After the CCR block, on the way towards the observable amide proton magnetization, we evolve the chemical shifts of $\textrm{C}^\alpha _{( i-1)}$, $\textrm{C}'_{( i-1)}$ and $\textrm{N}_{(i)}$, where only $\textrm{C}^\alpha _{( i-1)}$ is measured in constant-time mode (the CT length of 55 ms provides not only a proper evolution of the scalar coupling of $\textrm{C}^\alpha _{( i-1)}$with nitrogen and carbonyl carbon, but also a minimization of the coupling with C$^\beta$). Here the final steps of the coherence transfer are shown:

$$\begin{aligned} &4\textrm{N}_{z(i-1)}\textrm{C}^{\alpha }_{z(i-1)}\textrm{N}_\mathrm{{z(i)}}+ [16\textrm{H}^\textrm{N}_{z(i-1)}\textrm{N}_{z(i-1)}\textrm{C}^{\alpha }_{z(i-1)}\textrm{H}^\textrm{N}_{z(i)}\textrm{N}_\mathrm{{z(i)}}]\\&\quad\xrightarrow [J_{C^{\alpha }C'}]{^{1}J_{C^{\alpha }N},^{2}J_{C^{\alpha }N}} 2\textrm{C}^{\alpha }_{z(i-1)}\textrm{C}'_{z(i-1)}\quad+\\&\quad+ [8\textrm{H}^\textrm{N}_{z(i-1)}\textrm{C}^{\alpha }_{z(i-1)}\textrm{C}'_{z(i-1)}\textrm{H}^\textrm{N}_{z(i)}] \xrightarrow [J_{C'C^{\alpha }}]{J_{C'N}} \\&\quad\xrightarrow [J_{C'C^{\alpha }}]{J_{C'N}} 2\textrm{C}'_{z(i-1)}\textrm{N}_\mathrm{{z(i)}}\quad +\\&\quad+ [8\textrm{H}^\textrm{N}_{z(i-1)}\textrm{C}'_{z(i-1)}\textrm{N}_\mathrm{{z(i)}}\textrm{H}^\textrm{N}_{z(i)}] \xrightarrow [{J_{NC'}}]{J_{NH}} \\&\quad\xrightarrow [{J_{NC'}}]{J_{NH}} 2\textrm{H}^\textrm{N}_{z(i)}\textrm{N}_\mathrm{{z(i)}}+ [2\textrm{H}^\textrm{N}_{z(i-1)}\textrm{N}_\mathrm{{z(i)}}]\xrightarrow []{J_{HN}}\\&\quad\xrightarrow []{J_{HN}} \textrm{H}^\textrm{N}_{x,y(i)}+ [ \mathrm{multiple-quantum\quad coherence}] \end{aligned}$$

#### Experiment 4. $\textrm{H}^\textrm{N}_{(\textrm{i})}{\textrm{H}^{\alpha }_{(i-1)}}$ DD–$\textrm{C}'_{( i-1)}$ CSA

The fourth experiment measures the CCR effect between $\textrm{H}^\textrm{N}_{(\textrm{i})}{\textrm{H}^{\alpha }_{(i-1)}}$ DD and $\textrm{C}'_{( i-1)}$ CSA. From the peak ${\textrm{H}_{(\textrm{i})}-\textrm{N}_{(\textrm{i})}}$ – $\textrm{C}'_{( i-1)}$ – $\textrm{C}^\alpha _{( i-1)}$ we get information on ${\psi _{(i-1)}}$. This pulse sequence was published in 2020 (Kauffmann et al. 2020); please refer to this article for a full description.

#### Experiment 5. $${\textrm{C}^\alpha _{(i-1)}}$$$${\textrm{H}^\alpha _{(i-1)}}$$ DD–$$\textrm{C}'_{( i-1)}$$ CSA

**Fig. 6:**
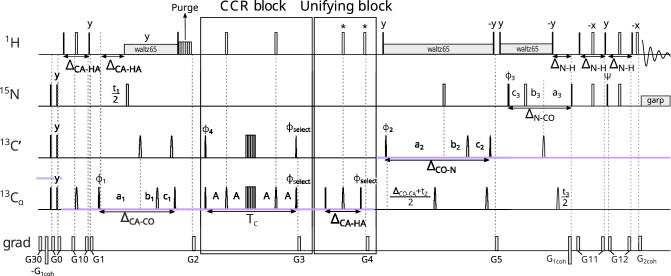
Pulse sequence of the experiment 5 ($\textrm{C}^{\alpha }_{(i-1)}\textrm{H}^{\alpha }_{(i-1)}$ DD–$\textrm{C}'_{(i-1)}$ CSA CCR). The evolution of ${\textrm{C}^{\alpha }}$ is in (semi)constant-time mode: $a_1=(\varDelta _{\mathrm{CA-CO}}+t_1)/2, \ b_1=t_1(1-\varDelta _{\mathrm{CA-CO}})/(2t^{max}_{1}), \ c_1=\varDelta _{\mathrm{CA-CO}}(1-t_1)/(2t^{max}_{1})$, C$'$ evolution is in (semi)constant-time mode: $a_2=(\varDelta _{\mathrm{CO-N}}+t_2)/2, \ b_2=t_2(1-\varDelta _{\mathrm{CO-N}})/(2t^{max}_{2}), \ c_2=\varDelta _{\mathrm{CO-N}}(1-t_2)/(2t^{max}_{2})$ and N evolution is in (semi)constant-time mode: $a_3=(\varDelta _{\mathrm{N-CO}}+t_3)/2, \ b_3=t_3(1-\varDelta _{\mathrm{N-CO}})/(2t^{max}_{3}), \ c_3=\varDelta _{\mathrm{N-CO}}(1-t_3)/(2t^{max}_{3})$. The semi-constant (Grzesiek and Bax 1993) or constant time mode is chosen automatically, based on the number of sampling points and spectral width in a given dimension. The delays were set as follows: $A = \textrm{T}_{c}/4$, $\varDelta _{\mathrm{N-H}}$ = 5.4 ms, $\varDelta _{\mathrm{N-CO}}$ = 33 ms, $\varDelta _{\mathrm{CO-N}}$ = 29 ms, $\varDelta _{\mathrm{CO-CA}}$ = 9.1 ms, $\varDelta _{\mathrm{CA-CO}}$ = 6.2 ms, and $\varDelta _{\mathrm{CA-HA}}$ = 3.2 ms. Total CCR rate evolution time is set as $\textrm{T}_{c}$ = 28.6 ms. Unless noted explicitly, pulse phases are set to x. Phase $\phi _{select}$ depends on the version of the experiment: for reference experiment x, for transfer experiment y. The value of individual phase cycles are $\phi _{1}=x,-x$, $\phi _{2}=2(x),2(-x)$, $\phi _{3}=x$, $\phi _{4}=4(x),4(y),4(-x),4(-y)$, $\psi =y$, and receiver $\phi _{r}=\phi _{1}+\phi _{2}+\phi _{4}$. Quadrature detection is achieved by States-TPPI (Marion et al. 1989) for ${\textrm{C}^{\alpha }}$ (change of $\phi _{1}$ phase) and C$'$ (change of $\phi _{2}$ phase) evolutions, and echo-antiecho (Kay et al. 1992; Schleucher et al. 1993) for N evolution (change of $\phi _{3}$ and $\psi$ phases and change of $G_{2coh}$ amplitude sign). Selective pulses for C$'$ and ${\textrm{C}^{\alpha }}$ spins were Q3 (for 180$^\circ$ pulses) and Q5 (for 90$^\circ$ pulses) (Emsley and Bodenhausen 1992). The pulse labelled by a star ("*") was executed only in the transfer version of the experiment. Rectangles represent hard pulses while rounded cones symbolize shaped pulses; the empty ones are 180$^\circ$ pulses and the filled ones are 90$^\circ$ pulses. Gradients with numbers from 1 to 12 are cleaning gradients. Gradients $G_{1coh}$ and $G_{2coh}$ are used for coherence selection in echo-antiecho quadrature detection. Proton decoupling is performed with composite pulse scheme waltz65 (Zhou et al. 2007) and nitrogen decoupling during acquisition is performed with composite pulse scheme garp (Shaka et al. 1985). The CCR and unifying blocks are indicated by rectangle boxes

The fifth experiment measures the CCR effect between $\textrm{C}^{\alpha }_{(i-1)}\textrm{H}^{\alpha }_{(i-1)}$ DD and $\textrm{C}'_{(i-1)}$ CSA. From the peak ${\textrm{H}^{\textrm{N}}_{(\textrm{i})}}-\textrm{N}_{(\textrm{i})}$ – $\textrm{C}'_{( i-1)}$ – $\textrm{C}^\alpha _{( i-1)}$ we get information on the ${\psi _{(i-1)}}$ angle. The first measurement of this rate was made by Yang et al. (1997, 1998) in a 3D J-resolved HN(CO)CA experiment, where the CCR rates were calculated using the intensities of zero quantum/double quantum peaks. Later, Chiarparin et al. (1999) carried out a 2D quantitative gamma experiment (without carbon evolution) with shifting pulses in the CCR block, leading to an identical form of coherence at the end of the CCR block in both reference and transfer versions. Unlike these experiments, our experiment is not ‘out-and-back’, and the coherence transfer starts from ${\textrm{H}^{\alpha }}$ rather than ${\textrm{H}^\textrm{N}}$ (see Fig. 6):

$$\begin{aligned} \textrm{H}^{\alpha }_{z(i-1)}\xrightarrow []{J_{C^{\alpha }H^{\alpha }}} 2\textrm{H}^{\alpha }_{z(i-1)}\textrm{C}^{\alpha }_{z(i-1)}\xrightarrow [J_{C^{\alpha }H^{\alpha }}]{J_{C^{\alpha }C'}} 2\textrm{C}^{\alpha }_{z(i-1)}\textrm{C}'_{z(i-1)}\Rightarrow \text {CCR block} \end{aligned}$$

**Fig. 7:**
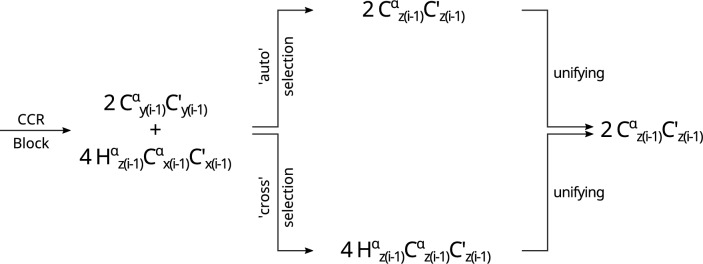
Scheme showing the difference in coherence transfer between transfer and reference version of Experiment 5 (${\textrm{C}^\alpha _{(i-1)}} {\textrm{H}^\alpha _{(i-1)}}$ DD - ${\textrm{C}'_{(i-1)}}$ CSA)

During the second INEPT, the (semi)constant-time evolution of ${\textrm{C}^{\alpha }}$ takes place. To minimize the effect of coherence loss due to C$^\alpha$-C$^\beta$ scalar coupling evolution, 10 ms of evolution time should not be exceeded. Because this experiment used ${\textrm{H}^{\alpha }}$ excitation, we add a purge pulse (as in Experiment 3 ($\textrm{N}_{(i-1)}\textrm{H}^{\textrm{N}}_{(i-1)}$ DD–$\textrm{N}_{(i)}\textrm{H}^{\textrm{N}}_{(i)}$ DD)) to crush water magnetization. The CCR block contains only the evolution of CCR $\textrm{C}^{\alpha }_{(i-1)}\textrm{H}^{\alpha }_{(i-1)}$ DD–$\textrm{C}'_{(i-1)}$ CSA (the unwanted evolution of $\textrm{C}^{\alpha }_{(i-1)}$ CSA - $\textrm{C}'_{(i-1)}$ CSA was refocused during the CCR block by the 180$^{\circ }$ pulses acting on alpha carbon nuclei). The initial 2$\textrm{C}^{\alpha }_{y(i-1)}$ $\textrm{C}'_{y(i-1)}$ coherence is partially converted into 4$\textrm{H}^{\alpha }_{z(i-1)}$ $\textrm{C}^{\alpha }_{x(i-1)}$ $\mathrm{C'}_{x(i-1)}$ coherence, so we can use different pulse phases to select each component: The x-pulse allows us to observe the unchanged coherence, while the y-pulse allows us to observe the component originating from the CCR evolution. To minimize the loss of coherence due to the ${\textrm{C}^{\alpha }-\textrm{C}^{\beta }}$ J-coupling evolution, the overall evolution time is fixed at 28.6 ms. During the unifying block, the 4$\textrm{H}^{\alpha }_{z(i-1)}$ $\textrm{C}^{\alpha }_{y(i-1)}$ $\textrm{C}'_{z(i-1)}$ coherence of the transfer version is transferred back to 2$\textrm{C}^{\alpha }_{x(i-1)}$ $\textrm{C}'_{z(i-1)}$, and in the reference version the 2$\textrm{C}^{\alpha }_{y(i-1)}$ $\textrm{C}'_{z(i-1)}$ coherence is not changed (see Fig. 7). The second 180$^\circ$ pulse acting on $^1$H (at the end of the transfer version of the unifying block) keeps the water magnetization along +z.

In the steps after the CCR block and unifying block, we evolve the chemical shifts of $\textrm{C}'_{( i-1)}$ and $\textrm{N}_{(i)}$. These steps are shown below:

$$\begin{aligned} 2\textrm{C}^{\alpha }_{z(i-1)}\textrm{C}'_{z(i-1)}\xrightarrow [J_{C'C^{\alpha }}]{J_{C'N}} 2\textrm{C}'_{z(i-1)}\textrm{N}_\mathrm{{z(i)}}\xrightarrow [{J_{NC'}}]{J_{NH}} 2\textrm{H}^\textrm{N}_{z(i)}\textrm{N}_\mathrm{{z(i)}}\xrightarrow []{J_{HN}} \textrm{H}^\textrm{N}_{x,y(i)}\end{aligned}$$

#### Experiment 6. $\textrm{C}^{\alpha }_{(i)}\textrm{H}^{\alpha }_{(i)}$ DD–$\textrm{C}'_{(i-1)}$ CSA

**Fig. 8:**
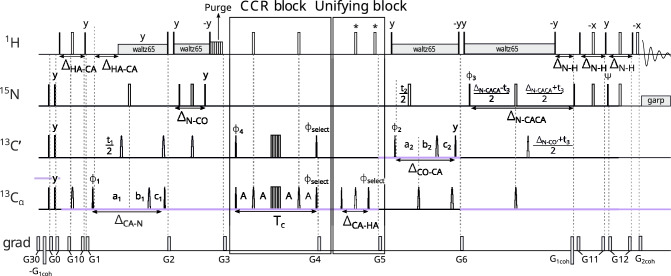
Pulse sequence of the experiment 6 ($\textrm{C}^{\alpha }_{(i)}\textrm{H}^{\alpha }_{(i)}$ DD–$\textrm{C}'_{(i-1)}$ CSA CCR). The evolution of ${\textrm{C}^{\alpha }}$ is in (semi)constant-time mode: $a_1=(\varDelta _{\mathrm{CA-N}}+t_1)/2, \ b_2=t_1(1-\varDelta _{\mathrm{CA-N}})/(2t^{max}_{1}), \ c_2=\varDelta _{\mathrm{CA-N}}(1-t_1)/(2t^{max}_{1})$, C$'$ evolution is in (semi)constant-time mode: $a_2=(\varDelta _{\mathrm{CO-CA}}+t_2)/2, \ b_2=t_2(1-\varDelta _{\mathrm{CO-CA}})/(2t^{max}_{2}), \ c_2=\varDelta _{\mathrm{CO-CA}}(1-t_2)/(2t^{max}_{2})$ and N evolution is in constant-time mode. The semi-constant (Grzesiek and Bax 1993) or constant time mode is chosen automatically, based on the number of sampling points and spectral width in a given dimension. The delays were set as follows: $A = \textrm{T}_{c}/4$, $\varDelta _{\mathrm{N-H}}$ = 5.4 ms, $\varDelta _{\mathrm{N-CO'}}$ = 33.5 ms, $\varDelta _{\mathrm{N-CACA}}$ = 50 ms, $\varDelta _{\mathrm{N-CO}}$ = 31 ms, $\varDelta _{\mathrm{CO-CA}}$ = 9.1 ms, $\varDelta _{\mathrm{CA-N}}$ = 27.2 ms, and $\varDelta _{\mathrm{CA-HA}}$ = 3.4 ms. Total CCR rate evolution time is set as $\textrm{T}_{c}$ = 28.6 ms. Unless noted explicitly, pulse phases are set to x. Phase $\phi _{select}$ depends on the version of the experiment: for reference experiment x, for transfer experiment y. The value of individual phase cycles are $\phi _{1}=x,-x$, $\phi _{2}=2(x),2(-x)$, $\phi _{3}=x$, $\phi _{4}=4(x),4(y),4(-x),4(-y)$, $\psi =y$, and receiver $\phi _{r}=\phi _{1}+\phi _{2}+\phi _{4}$. Quadrature detection is achieved by States-TPPI (Marion et al. 1989) for ${\textrm{C}^{\alpha }}$ (change of $\phi _{1}$ phase) and C$'$ (change of $\phi _{2}$ phase) evolutions, and echo-antiecho (Kay et al. 1992; Schleucher et al. 1993) for N evolution (change of $\phi _{3}$ and $\psi$ phases and change of $G_{2coh}$ amplitude sign). Selective pulses for C$'$ and ${\textrm{C}^{\alpha }}$ spins were Q3 (for 180$^\circ$ pulses) and Q5 (for 90$^\circ$ pulses) (Emsley and Bodenhausen 1992).The pulse labelled by a star ("*") was executed only in the transfer version of the experiment. Rectangles represent hard pulses while rounded cones symbolize shaped pulses; the empty ones are 180$^\circ$ pulses and the filled ones are 90$^\circ$ pulses. Gradients with numbers from 1 to 12 are cleaning gradients. Gradients $G_{1coh}$ and $G_{2coh}$ are used for coherence selection in echo-antiecho quadrature detection. Proton decoupling is performed with composite pulse scheme waltz65 (Zhou et al. 2007) and nitrogen decoupling during acquisition is performed with composite pulse scheme garp (Shaka et al. 1985). The CCR and unifying blocks are indicated by rectangle boxes

The sixth experiment measures the CCR effect between $\textrm{C}^{\alpha }_{(i)}\textrm{H}^{\alpha }_{(i)}$ DD and $\textrm{C}'_{(i-1)}$ CSA. From the peak $\textrm{H}^{\textrm{N}}_{(\textrm{i})}-\textrm{N}_{(\textrm{i})}$ – $\textrm{C}'_{( i-1)}$ – $\textrm{C}^\alpha _{(\textrm{i})}$ we get information on the ${\phi _{(\textrm{i})}}$ angle. This CCR rate was previously measured by Kloiber and Konrat (2000a) in a 3D quantitative gamma experiment. As the pulse sequence was based on the HNCA experiment, the spectra contained two peaks per residue, ${\textrm{H}_{(\textrm{i})}-\textrm{N}_{(\textrm{i})}}$ – $\textrm{C}^\alpha _{(\textrm{i})}$ and ${\textrm{H}_{(\textrm{i})}-\textrm{N}_{(\textrm{i})}}$ – $\textrm{C}^\alpha _{( i-1)}$, which made it possible to calculate two CCR rates ($\textrm{C}^{\alpha }_{(i-1)}\textrm{H}^{\alpha }_{(i-1)}$ DD–$\textrm{C}'_{(i-1)}$ CSA and $\textrm{C}^{\alpha }_{(i)}\textrm{H}^{\alpha }_{(i)}$ DD–$\textrm{C}'_{(i-1)}$ CSA ) from a single experiment. However, to reduce peak overlap, we decided to modify the coherence transfer pathway and obtain a single peak per residue. The pulse sequence of our experiment is shown in Fig. 8. As in Experiment 5 (${\textrm{C}^\alpha _{(i-1)}} {\textrm{H}^\alpha _{(i-1)}}$ DD–${\textrm{C}'_{(i-1)}}$ CSA), the pulse sequence starts with ${\textrm{H}^{\alpha }}$ excitation:

$$\begin{aligned} &\textrm{H}^{\alpha }_{z(i)}\xrightarrow []{J_{C^{\alpha }H^{\alpha }}} 2\textrm{H}^{\alpha }_{z(i)}\textrm{C}^{\alpha }_{z(i)} \\& \xrightarrow [J_{C^{\alpha }H^{\alpha }}]{^1J_{C^{\alpha }N}, ^2J_{C^{\alpha }N}} \quad 2 \textrm{N}_\mathrm{{z(i)}}\textrm{C}^{\alpha }_{z(i)}+ [2 \textrm{C}^{\alpha }_{z(i)}\textrm{N}_{z(i+1)}] \xrightarrow []{J_{NC'}} \\&\quad \xrightarrow []{J_{NC'}} 4\textrm{C}'_{z(i-1)}\textrm{N}_\mathrm{{z(i)}}\textrm{C}^{\alpha }_{z(i)}+ [4 \textrm{C}'_{z(i)}\textrm{C}^{\alpha }_{z(i)}\textrm{N}_{z(i+1)}] \\&\quad \Rightarrow \text {CCR block} \end{aligned}$$

and during the second transfer the (semi-)constant time evolution of ${\textrm{C}^{\alpha }}$ occurs. It is recommended not to exceed the CT length (27.2 ms) to avoid peak splitting due to C$^\alpha$–C$^\beta$ scalar coupling.

As in Experiment 5 (${\textrm{C}^\alpha _{(i-1)}} {\textrm{H}^\alpha _{(i-1)}}$ DD–${\textrm{C}'_{(i-1)}}$ CSA), after the second INEPT block, where the constant time/semi-constant time evolution of C$^\alpha$ nuclei takes place, we add a purge pulse in order to reduce the water signal. Presaturation can be used for the same purpose. Before the CCR block, we have two coherences: 4$\textrm{C}'_{z(i-1)}$ $\textrm{N}_\mathrm{{z(i)}}$ $\textrm{C}^{\alpha }_{z(i)}$ and 4$\textrm{C}'_{z(i)}$ $\textrm{C}^{\alpha }_{z(i)}$ $\textrm{N}_{z(i+1)}$. Only the former contains information on the desired CCR rate; the latter is not transferred to observable magnetization and in the coherence transfer schemes it is shown in squared brackets. During the CCR block, the initial 4$\textrm{C}'_{y(i-1)}$ $\textrm{N}_\mathrm{{z(i)}}$ $\textrm{C}^{\alpha }_{y(i)}$ coherence evolves only due to $\textrm{C}^{\alpha }_{(i)}\textrm{H}^{\alpha }_{(i)}$ DD–$\textrm{C}'_{(i-1)}$ CSA interference. This leads to a partial convertion into 8$\mathrm{C'}_{x(i-1)}$ $\textrm{N}_\mathrm{{z(i)}}$ $\textrm{C}^{\alpha }_{x(i)}$ $\textrm{H}^{\alpha }_{z(i)}$ coherence, so we can use different pulse phases to select each component—the x-pulse allows us to observe the unchanged coherence, while the y-pulse allows us to observe the component originating from CCR evolution. To minimize the loss of sensitivity due to the ${\textrm{C}^{\alpha }-\textrm{C}^{\beta }}$ J-coupling evolution, we set the overall evolution time permanently to 28.6 ms. During the unifying block, the 8$\textrm{C}'_{z(i-1)}$ $\textrm{N}_\mathrm{{z(i)}}$ $\textrm{C}^{\alpha }_{y(i)}$ $\textrm{H}^{\alpha }_{z(i)}$ coherence of the transfer version is transferred back to 4$\textrm{C}'_{z(i-1)}$ $\textrm{N}_\mathrm{{z(i)}}$ $\textrm{C}^{\alpha }_{x(i)}$. The unwanted coherence (entering the CCR block as 4$\textrm{C}'_{z(i)}$ $\textrm{C}^{\alpha }_{z(i)}$ $\textrm{N}_{z(i+1)}$) behaves analogously. Similarly to experiment 5, the unwanted CCR evolution of $\textrm{C}'_{(i-1)}$ CSA–$\textrm{C}^{\alpha }_{(i)}$ CSA was refocused during the CCR block by the 180$^{\circ }$ pulses acting on alpha carbon nuclei.

In the following steps, we evolve the chemical shifts of $\textrm{C}'_{( i-1)}$ and $\textrm{N}_{(i)}$. The evolution of the $\textrm{N}_{(i)}$ chemical shift is executed in constant-time mode, as the INEPT delay is very long (50 ms) and there is no need to implement a semi-constant time evolution option. This is the corresponding coherence transfer:

$$\begin{aligned}&4\textrm{C}'_{z(i-1)}\textrm{N}_\mathrm{{z(i)}}\textrm{C}^{\alpha }_{z(i)}+ [4 \textrm{C}'_{z(i)}\textrm{C}^{\alpha }_{z(i)}\textrm{N}_{z(i+1)}] \xrightarrow []{J_{C'C^{\alpha }}}\\&\quad \xrightarrow []{J_{C'C^{\alpha }}} 8\textrm{C}'_{z(i-1)}\textrm{C}^{\alpha }_{z(i-1)}\textrm{N}_\mathrm{{z(i)}}\textrm{C}^{\alpha }_{z(i)}+ [2 \textrm{C}'_{z(i)}\textrm{N}_{z(i+1)}] \\&\quad \xrightarrow [J_{NH},J_{NC'}]{^1J_{NC^{\alpha }},^2J_{NC^{\alpha }}} 2\textrm{H}^\textrm{N}_{z(i)}\textrm{N}_\mathrm{{z(i)}}+ [8 \textrm{C}'_{z(i)}\textrm{C}^{\alpha }_{z(i)}\textrm{N}_{z(i+1)}\textrm{C}^{\alpha }_{z(i+1)}] \\&\quad \xrightarrow []{J_{HN}} \textrm{H}^\textrm{N}_{x,y(i)}+ [\mathrm{multiple-quantum\quad coherence}] \end{aligned}$$

#### Experiment 7. $\textrm{N}_{(i)}\textrm{H}^{\textrm{N}}_{(i)}$ DD–$\textrm{C}'_{(i)}$ CSA

The seventh experiment measures the CCR effect between $\textrm{N}_{(i)}\textrm{H}^{\textrm{N}}_{(i)}$ DD and $\textrm{C}'_{(i)}$ CSA. From the peak ${\textrm{H}^{\textrm{N}}_{(\textrm{i})}-\textrm{N}_{(\textrm{i})}}$ – $\textrm{C}'_{(\textrm{i})}$ – $\textrm{C}^\alpha _{(\textrm{i})}$ we get information on the ${\psi _{(\textrm{i})},\phi _{(\textrm{i})}}$ angles. Previously, this CCR rate was measured in a 3D J-resolved HN(CA)CO experiment (Kloiber and Konrat 2000b). Combining separately acquired cosine and sine modulations of $J_{NH}$ coupling leads to two separate spectra, one with up-field and the other with down-field components. Stanek et al. (2013) designed a 4D version of this experiment, which provides one spectrum with two J-resolved peaks giving information on two CCR rates: $\textrm{N}_{(i)}\textrm{H}^{\textrm{N}}_{(i)}$ DD–$\textrm{C}'_{(i)}$ CSA and $\textrm{N}_{(i)}\textrm{H}^{\textrm{N}}_{(i)}$ DD–$\textrm{C}'_{(i-1)}$ CSA. The latter rate provides information on the $\omega$ peptide bond dihedral angle, which is typically close to 180 degrees.

In both approaches described above, we have to deal with two peaks per residue. We therefore optimize the coherence transfer to provide a single peak per residue:

$$\begin{aligned} &{\textrm{H}}^{N}_{z(i+1)}\xrightarrow []{J_{NH}} 2{\textrm{H}}^{N}_{z(i+1)}\textrm{N}_{z(i+1)}\xrightarrow [J_{NH}]{J_{NC'}} 2\textrm{C}'_{z(i)}\textrm{N}_{z(i+1)}\xrightarrow [J_{NC'}]{{J_{C'{C^{\alpha }}}}} \\&\quad \xrightarrow [J_{NC'}]{J_{C'C^{\alpha }}} 4\textrm{C}^{\alpha }_{z(i)}\textrm{C}'_{z(i)}\textrm{N}_{z(i+1)}\xrightarrow [^2J_{NC^{\alpha }}]{^1J_{NC^{\alpha }}} 4\textrm{N}_\mathrm{{z(i)}}\textrm{C}^{\alpha }_{z(i)}\textrm{C}'_{z(i)}\\ &\quad \Rightarrow \text {CCR block} \end{aligned}$$

**Fig. 9:**
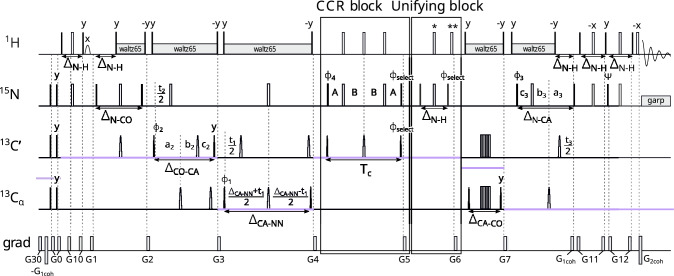
Pulse sequence of the experiment 7 ($\textrm{N}_{(i)}\textrm{H}^{\textrm{N}}_{(i)}$ DD–$\textrm{C}'_{(i)}$ CSA CCR). The evolution of ${\textrm{C}^{\alpha }}$ is in constant-time mode, C$'$ evolution is in (semi)constant-time mode: $a_2=(\varDelta _{\mathrm{CO-CA}}+t_2)/2, \ b_2=t_2(1-\varDelta _{\mathrm{CO-CA}})/(2t^{max}_{2}), \ c_2=\varDelta _{\mathrm{CO-CA}}(1-t_2)/(2t^{max}_{2})$ and N evolution is in (semi)constant-time mode: $a_3=(\varDelta _{\mathrm{N-CA}}+t_3)/2, \ b_3=t_3(1-\varDelta _{\mathrm{N-CA}})/(2t^{max}_{3}), \ c_3=\varDelta _{\mathrm{N-CA}}(1-t_3)/(2t^{max}_{3})$. The semi-constant (Grzesiek and Bax 1993) or constant time mode is chosen automatically, based on the number of sampling points and spectral width in a given dimension. The delays were set as follows: $A = (\textrm{T}_{c}+p14)/4-p21/\pi$, $B = (\textrm{T}_{c}-p14)/4+p21/\pi$ (where *p*14 is the length of 180$^\circ$ carbon shaped pulse and *p*21 is the length of 90$^\circ$ nitrogen hard pulse), $\textrm{T}_{c}$ = 28 ms, $\varDelta _{\mathrm{N-H}}$ = 5.4 ms, $\varDelta _{\mathrm{N-CO}}$ = 31 ms, $\varDelta _{\mathrm{N-CA}}$ = 28 ms, $\varDelta _{\mathrm{CA-NN}}$ = 55 ms, $\varDelta _{\mathrm{CO-CA}}$ = 9.1 ms, $\varDelta _{\mathrm{CA-CO}}$ = 6 ms. Unless noted explicitly, pulse phases are set to x. Phase $\phi _{select}$ depends on the version of the experiment: for reference experiment x, for transfer experiment y. The value of individual phase cycles are $\phi _{1}=x,-x$, $\phi _{2}=2(x),2(-x)$, $\phi _{3}=x$, $\phi _{4}=x$, $\psi =y$ and receiver $\phi _{r}=\phi _{1}+\phi _{2}+\phi _{4}$. Quadrature detection is achieved by States-TPPI (Marion et al. 1989) for ${\textrm{C}^{\alpha }}$ (change of $\phi _{1}$ phase) and C$'$ (change of $\phi _{2}$ phase) evolutions, and echo-antiecho (Kay et al. 1992; Schleucher et al. 1993) for N evolution (change of $\phi _{3}$ and $\psi$ phases and change of $G_{2coh}$ amplitude sign). Selective pulses for C$'$ and ${\textrm{C}^{\alpha }}$ spins were Q3 (for 180$^\circ$ pulses) and Q5 (for 90$^\circ$ pulses) (Emsley and Bodenhausen 1992). The pulse labelled by a one star ("*") was executed only in the transfer version and the pulse labelled by a two star ("**") was executed only in the reference version of the experiment. Rectangles represent hard pulses while rounded cones symbolize shaped pulses; the empty ones are 180$^\circ$ pulses and the filled ones are 90$^\circ$ pulses. Gradients with numbers from 1 to 12 are cleaning gradients. Gradients $G_{1coh}$ and $G_{2coh}$ are used for coherence selection in echo-antiecho quadrature detection. Proton decoupling is performed with composite pulse scheme waltz65 (Zhou et al. 2007) and nitrogen decoupling during acquisition is performed with composite pulse scheme garp (Shaka et al. 1985). The CCR and unifying blocks are indicated by rectangle boxes

In our pulse sequence, the evolution of $\textrm{C}'_{(\textrm{i})}$ and $\textrm{C}^\alpha _{(\textrm{i})}$ occurs before the CCR block (see Fig. 9). We execute the evolution of the $\textrm{C}^\alpha _{(\textrm{i})}$ chemical shift in constant-time mode because the INEPT delay is very long (55 ms) and there is no need to use the semi-constant time evolution option. Such length of the delay provides also proper handling of C$^\alpha$–C$^\beta$ scalar coupling.

During the CCR block, only one CCR rate ($\textrm{N}_{(i)}\textrm{H}^{\textrm{N}}_{(i)}$ DD–$\textrm{C}'_{(i)}$ CSA ) is evolving: The initial 4$\textrm{N}_{y(i)}$ $\textrm{C}^{\alpha }_{z(i)}$ $\textrm{C}'_{y(i)}$ coherence is partially converted into 8$\textrm{H}^\textrm{N}_{z(i)}$ $\textrm{N}_{x(i)}$ $\textrm{C}^{\alpha }_{z(i)}$ $\textrm{C}'_{x(i)}$ coherence, so we can use different phases of pulses to select each component—the x-pulses allow us to observe the unchanged coherence, while the y-pulses allow us to observe the component originating from CCR evolution. During the unifying block, the 8$\textrm{H}^\textrm{N}_{z(i)}$ $\textrm{N}_\mathrm{{z(i)}}$ $\textrm{C}^{\alpha }_{z(i)}$ $\textrm{C}'_{z(i)}$ operator is converted back to 4$\textrm{N}_\mathrm{{z(i)}}$ $\textrm{C}^{\alpha }_{z(i)}$ $\textrm{C}'_{z(i)}$. The undesired evolution of $\textrm{N}_{(i)}$ CSA–$\textrm{C}'_{(i)}$ CSA was refocused during the CCR block by the 180$^\circ$ pulse acting on carbonyl carbon nuclei.

On the way back, we evolve the chemical shifts of $N_{(i)}$. This is the related coherence transfer pathway:

$$\begin{aligned} &4\textrm{N}_\mathrm{{z(i)}}\textrm{C}^{\alpha }_{z(i)}\textrm{C}'_{z(i)}\xrightarrow []{J_{C^{\alpha }C'}} 2\textrm{N}_\mathrm{{z(i)}}\textrm{C}^{\alpha }_{z(i)}\xrightarrow [J_{NH}]{^1J_{NC^{\alpha }},^2J_{NC^{\alpha }}}\\&\rightarrow 2 \textrm{H}^\textrm{N}_{z(i)}\textrm{N}_\mathrm{{z(i)}}+ [8\textrm{C}^{\alpha }_{z(i-1)}\textrm{H}^\textrm{N}_{z(i)}\textrm{N}_\mathrm{{z(i)}}\textrm{C}^{\alpha }_{z(i)}] \xrightarrow []{J_{NH}} \\&\xrightarrow []{J_{HN}} \textrm{H}^\textrm{N}_{x,y(i)}+ [4\textrm{C}^{\alpha }_{z(i-1)}\textrm{H}^\textrm{N}_{x,y(i)}\textrm{C}^{\alpha }_{z(i)}] \end{aligned}$$

In the INEPT block with nitrogen evolution, the evolution of the $J_{CA-N}$ scalar coupling partially leads to the creation of the unwanted term 8$\textrm{C}^{\alpha }_{z(i-1)}$ $\textrm{H}^\textrm{N}_{z(i)}$ $\textrm{N}_\mathrm{{z(i)}}$ $\textrm{C}^{\alpha }_{z(i)}$ (in the scheme above shown in squared brackets), which later evolves and enters the acquisition as anti-phase magnetization 4$\textrm{C}^{\alpha }_{z(i-1)}$ $\textrm{H}^\textrm{N}_{x,y(i)}$ $\textrm{C}^{\alpha }_{z(i)}$. In principle, during the acquisition time, this term could evolve into observable magnetization, but taking into account the small values of scalar coupling constants (amide proton with alpha carbon nuclei of the same and previous residues) as well as splitting pattern of the expected peak, it can be neglected.

#### Experiment 8. $\textrm{C}'_{(i-1)}$ CSA–$\textrm{C}'_{(i)}$ CSA

The last of our experiments is for measuring the CCR effect between $\textrm{C}'_{(i-1)}$ CSA and $\textrm{C}'_{(i)}$ CSA. From peak ${\textrm{H}^{\textrm{N}}_{(\textrm{i})}-\textrm{N}_{(\textrm{i})}}$ – $\textrm{C}'_{( i-1)}$ – $\textrm{C}^\alpha _{(\textrm{i})}$ we get information on the ${\psi _{(\textrm{i})}, \phi _{(\textrm{i})}}$ angle. In 2000, Skrynnikov et al. (2000) published an experiment for measuring this CCR rate using a double-quantum and a zero-quantum approach. Two spectra, acquired with different phases (x or y) of the first pulse in the CCR block, are added to provide a double-quantum spectrum, or subtracted to provide a zero-quantum spectrum. We then calculate the CCR rate using the following formula:

13 $$\begin{aligned} \varGamma = \frac{1}{2Tc}\cdot \ln {\frac{I_{DQ}}{I_{ZQ}}} \end{aligned}$$

where *Tc* is the time of CCR evolution, and $I_{DQ}$ and $I_{ZQ}$ are the peak heights in the double-quantum and zero-quantum versions, respectively.

**Fig. 10:**
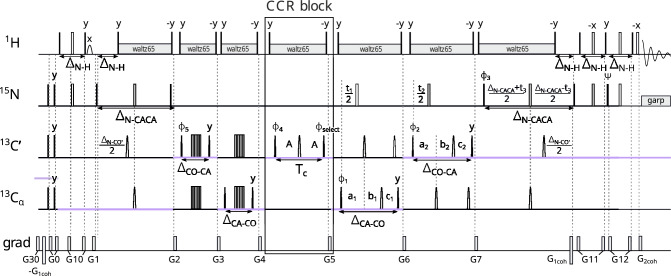
Pulse sequence of the experiment 8 ($\textrm{C}'_{(i-1)}$ CSA–$\textrm{C}'_{(i)}$ CSA CCR). The evolution of ${\textrm{C}^{\alpha }}$ is in (semi)constant-time mode: $a_1=(\varDelta _{\mathrm{CA-CO}}+t_1)/2, \ b_2=t_1(1-\varDelta _{\mathrm{CA-CO}})/(2t^{max}_{1}), \ c_2=\varDelta _{\mathrm{CA-CO}}(1-t_1)/(2t^{max}_{1})$, C$'$ evolution is in (semi)constant-time mode: $a_2=(\varDelta _{\mathrm{CO-CA}}+t_2)/2, \ b_2=t_2(1-\varDelta _{\mathrm{CO-CA}})/(2t^{max}_{2}), \ c_2=\varDelta _{\mathrm{CO-CA}}(1-t_2)/(2t^{max}_{2})$ and N evolution is in constant-time mode. The semi-constant (Grzesiek and Bax 1993) or constant time mode is chosen automatically, based on the number of sampling points and spectral width in a given dimension. The delays were set as follows: $A = \textrm{T}_{c}/2$, $\textrm{T}_{c}$ = 28 ms, $\varDelta _{\mathrm{N-H}}$ = 5.4 ms, $\varDelta _{\mathrm{N-CO'}}$ = 33.5 ms, $\varDelta _{\mathrm{N-CACA}}$ = 50 ms, $\varDelta _{\mathrm{CO-CA}}$ = 9.1 ms, and $\varDelta _{\mathrm{CA-CO}}$ = 6 ms. Unless noted explicitly, pulse phases are set to x. Phase $\phi _{select}$ depends on the version of the experiment: for reference experiment x, for transfer experiment y. The value of individual phase cycles are $\phi _{1}=x,-x$, $\phi _{2}=2(x),2(-x)$, $\phi _{3}=x$, $\phi _{4}=16(x),16(-x)$, $\phi _{5}=8(x),8(-x)$, $\psi =y$ and receiver $\phi _{r}=\phi _{1}+\phi _{2}+\phi _{5}$. Quadrature detection is achieved by States-TPPI (Marion et al. 1989) for ${\textrm{C}^{\alpha }}$ (change of $\phi _{1}$ phase) and C$'$ (change of $\phi _{2}$ phase) evolutions, and echo-antiecho (Kay et al. 1992; Schleucher et al. 1993) for N evolution (change of $\phi _{3}$ and $\psi$ phases and change of $G_{2coh}$ amplitude sign). Selective pulses for C$'$ and ${\textrm{C}^{\alpha }}$ spins were Q3 (for 180$^\circ$ pulses) and Q5 (for 90$^\circ$ pulses) (Emsley and Bodenhausen 1992). Rectangles represent hard pulses while rounded cones symbolize shaped pulses; the empty ones are 180$^\circ$ pulses and the filled ones are 90$^\circ$ pulses. Gradients with numbers from 1 to 12 are cleaning gradients. Gradients $G_{1coh}$ and $G_{2coh}$ are used for coherence selection in echo-antiecho quadrature detection. Proton decoupling is performed with composite pulse scheme waltz65 (Zhou et al. 2007) and nitrogen decoupling during acquisition is performed with composite pulse scheme garp (Shaka et al. 1985). The CCR and unifying blocks are indicated by rectangle boxes

In this approach there is no possibility of performing quadrature detection in the double-quantum or zero-quantum dimension, which results in twice the number of peaks. For this reason we designed the pulse sequence using a quantitative-gamma approach (see Fig. 10). The coherence transfer before the CCR block is as follows:

$$\begin{aligned} \textrm{H}^\textrm{N}_{z(i)}&\xrightarrow []{J_{NH}} 2\textrm{H}^\textrm{N}_{z(i)}\textrm{N}_\mathrm{{z(i)}}\\&\xrightarrow [J_{NH},J_{NC'}]{^{1}J_{NC^{\alpha }},^{2}J_{NC^{\alpha }}}8\textrm{C}^{\alpha }_{z(i-1)}\textrm{C}'_{z(i-1)}\textrm{N}_\mathrm{{z(i)}}\textrm{C}^{\alpha }_{z(i)}\xrightarrow []{J_{C'C^{\alpha }}} \\&\xrightarrow []{J_{C'C^{\alpha }}} 4\textrm{C}'_{z(i-1)}\textrm{N}_\mathrm{{z(i)}}\textrm{C}^{\alpha }_{z(i)}\\&\xrightarrow []{J_{C^{\alpha }C'}} 8\textrm{C}'_{z(i-1)}\textrm{N}_\mathrm{{z(i)}}\textrm{C}^{\alpha }_{z(i)}\textrm{C}'_{z(i)}\Rightarrow \text {CCR block} \end{aligned}$$

Here, the CCR block is very simple and only one CCR rate ($\textrm{C}'_{(i-1)}$ CSA–$\textrm{C}'_{(i)}$ CSA ) evolves: The initial 8$\textrm{C}'_{y(i-1)}$ $\textrm{N}_\mathrm{{z(i)}}$ $\textrm{C}^{\alpha }_{z(i)}$ $\textrm{C}'_{y(i)}$ coherence is partially converted into 8$\mathrm{C'}_{x(i-1)}$ $\textrm{N}_\mathrm{{z(i)}}$ $\textrm{C}^{\alpha }_{z(i)}$ $\textrm{C}'_{x(i)}$ coherence, so we can use different pulse phases to select each component—the x-pulse allows us to observe the unchanged coherence, while the y-pulse allows us to observe the component originating from the CCR evolution. In this pulse sequence there is no need to have a unifying block, as after the CCR block in both cases we get 8$\textrm{C}'_{z(i-1)}$ $\textrm{N}_\mathrm{{z(i)}}$ $\textrm{C}^{\alpha }_{z(i)}$ $\textrm{C}'_{z(i)}$.

On the way back, we evolve the chemical shifts of $\textrm{C}^\alpha _{(\textrm{i})}$, $\textrm{C}'_{( i-1)}$ and $\textrm{N}_{(i)}$. Although $\textrm{C}^\alpha _{(\textrm{i})}$ evolution is performed in a semi-constant time mode, it should not exceed 10 ms to avoid signal loss due to scalar coupling with C$^\beta$. We carry out the evolution of the $\textrm{N}_{(i)}$ chemical shift in constant-time mode, as the INEPT delay is very long (50 ms) and there is no need to implement the semi-constant time evolution option. The coherence transfer pathway of this fragment of the experiment is as follows:

$$\begin{aligned} & 8\textrm{C}'_{z(i-1)}\textrm{N}_\mathrm{{z(i)}}\textrm{C}^{\alpha }_{z(i)}\textrm{C}'_{z(i)}\xrightarrow []{J_{C^{\alpha }C'}} 4\textrm{C}'_{z(i-1)}\textrm{N}_\mathrm{{z(i)}}\textrm{C}^{\alpha }_{z(i)}\xrightarrow []{J_{C'C^{\alpha }}} \\&\quad \xrightarrow []{J_{C'C^{\alpha }}} 8\textrm{C}^{\alpha }_{z(i-1)}\textrm{C}'_{z(i-1)}\textrm{N}_\mathrm{{z(i)}}\textrm{C}^{\alpha }_{z(i)}\xrightarrow [J_{NH},J_{NC'}]{^1J_{NC^{\alpha }},^2J_{NC^{\alpha }}}\\&\quad \xrightarrow [J_{NH},J_{NC'}]{^1J_{NC^{\alpha }},^2J_{NC^{\alpha }}} 2\textrm{H}^\textrm{N}_{z(i)}\textrm{N}_\mathrm{{z(i)}}\xrightarrow []{J_{HN}} \textrm{H}^\textrm{N}_{x,y(i)}\end{aligned}$$

## Experimental

All experiments were validated on a 2 mM $^{13}$C, $^{15}$N-labeled, untagged Ubiquitin sample purchased from ASLA Biotech AB (Riga, Latvia). The protein was dissolved in 10 mM of potassium phosphate buffer with a pH = 6.5, with 0.05% sodium azide and 10% deuterium oxide. We carried out the experiments on a Bruker AVANCE III HD 800 MHz spectrometer equipped with a 5 mm TCI-HCN cryo-probe. The temperature was 298 K. The experimental parameters are shown in Table 2.

**Table 2:** Experimental parameters for all experiments

Exp	ni	${\textrm{C}^{\alpha }}$		C$'$		N		ver	ns	Time, h
		sw, Hz	$t_{max}$,s	sw, Hz	$t_{max}$,s	sw, Hz	$t_{max}$,s			
1	200	5600	0.028	3000	0.027	2300	0.022	Reference	4	2.8
								Transfer	24	18.8
2	200	5000	0.010	2200	0.0273	2300	0.0278	Reference	4	2.8
								Transfer	32	21.6
3	200	5000	0.010	2200	0.027	2300	0.028	Reference	4	2.8
								Transfer	32	22.0
4	200	5000	0.010	2200	0.017	2300	0.028	Reference	4	2.6
								Transfer	32	21.2
5	200	5000	0.010	2200	0.027	2300	0.028	Reference	4	2.6
								Transfer	32	20.8
6	200	5000	0.010	2200	0.027	2300	0.028	Reference	4	2.7
								Transfer	32	21.7
7	200	5000	0.0067	2200	0.0091	2300	0.028	Reference	4	2.7
								Transfer	32	21.6
8	200	5000	0.0067	2200	0.0091	2300	0.028	Reference	4	2.7
								Transfer	32	21.5

*exp* experiment number (see methods), *ni* number of hypercomplex data points in all indirectly-detected dimensions, *sw* spectrum width, $t_{max}$ maximum time of evolution in a given indirect dimension, *ver* version of experiment, *ns* number of scans, *time* time of experiment

The amide-proton selective pulses in Experiment 1 ($\textrm{C}^{\alpha }_{(i-1)}\textrm{H}^{\alpha }_{(i-1)}$ DD–$\textrm{N}_{(i)}\textrm{H}^{\textrm{N}}_{(i)}$ DD) and Experiment 2 ($\textrm{C}^{\alpha }_{(i)}\textrm{H}^{\alpha }_{(i)}$ DD–$\textrm{N}_{(i)}\textrm{H}^{\textrm{N}}_{(i)}$ DD) were defined within the pulse program using the Bruker tool WaveMaker (offset of 4.3 ppm, bandwidth of 3 ppm, reburp shape (Geen and Freeman 1991)).

### Data analysis

We recorded all experiments using non-uniform sampling (NUS) and processed them with the compressed sensing method (Kazimierczuk and Orekhov 2011) using the mddnmr software (Orekhov et al. 2004-2019). A total of 500 iterations were performed and the $\lambda$ parameter was set to 0.01, providing optimal quantitative results.

We displayed the spectra using the Sparky program (Lee et al. 2015). To draw up the peak lists and calculate the CCR rates, we developed two Python scripts. The first script reads the peak heights from the Sparky-format spectrum using a known peak list and adjusts each position to the locally highest point; during this step the peaks which are not visible in the reference spectrum are discarded. The second script calculates the CCR rates for each residue (except for the glycine residues and overlapping peaks) using previously obtained peak heights (Eq. 12). In Experiment 5 (${\textrm{C}^\alpha _{(i-1)}} {\textrm{H}^\alpha _{(i-1)}}$ DD–${\textrm{C}'_{(i-1)}}$ CSA) and Experiment 6 ($\textrm{C}^{\alpha }_{(i)}\textrm{H}^{\alpha }_{(i)}$ DD–$\textrm{C}'_{(i-1)}$ CSA) only, the CCR rates were multiplied by $-1$, which is consistent with the data predicted by the structure.

We calculated the uncertainty of the experimental CCR rates on the basis of the thermal noise of the reference and the transfer spectrum of respective CCR experiments, using the uncertainty propagation method.

In order to compare the measured CCR rates to the previously published experimental data, we calculated the structure-predicted CCR rates for ubiquitin separately for each of the three structures deposited in the Protein Data Bank (PDB): 1D3Z (Cornilescu et al. 1998) (based on NOESY data), 2NR2 (Richter et al. 2007) (based on NOESY data), and 6V5D (Smith et al. 2020) (based on NOESY and residual dipolar coupling data, RDC). To calculate CCR rates involving the CSA relaxation mechanism, we had to make some assumptions about the tensor components: We set them in line with Cisnetti et al. (2004). We also set the correlation times based on Ceccolini et al. (2024) (calculated from the $\textrm{NH}^{\textrm{N}}_{(i)}$ DD–$\textrm{N}_{(i)}$ CSA CCR rates).

All the structures used contain more than one submitted conformer (1D3Z: 10, 2NR2: 144, 6V5D: 176). For each residue, we calculated the structure-predicted CCR rate as the mean value of the rates calculated for individual conformers. In the comparison of the experimentally obtained CCR rates with those back-calculated from the PDB structures (11) we reported the mean value as well as the standard deviation (grey horizontal lines). The standard deviation does not correspond to any experimental uncertainty, but it reflects the variability of the calculated structures. For rates involving the CSA mechanism, we considered also the variability of the back-calculated CCR rates originating from possible variations of the CSA tensor components (estimated in line with Cisnetti et al. (2004)) and we found that they were much smaller. Therefore we did not subject them to further analysis.

We calculated dihedral angle distributions using all the CCR rates measured, applying the maximum entropy approach described in Kauffmann et al. (2020). The structure-based angles were extracted from the ubiquitin structures deposited in PDB: In addition to 1D3Z, 2NR2, and 6V5D, which were used previously, we used a crystalographical structure 1UBI (Ramage et al. (1994), 1 submitted conformer).

## Results and discussion

The proposed set of experiments was validated using a folded protein, ubiquitin, for which the structural information is available. Ubiquitin consists of 76 amino acid residues, including three proline and six glycine residues. As all of our experiments are based on the detection of amide proton, no peaks of proline residues were observed. Nonetheless, four experiments (Experiment 1 ($\textrm{C}^{\alpha }_{(i-1)}\textrm{H}^{\alpha }_{(i-1)}$ DD–$\textrm{N}_{(i)}\textrm{H}^{\textrm{N}}_{(i)}$ DD), Experiment 3 ($\textrm{N}_{(i-1)}\textrm{H}^{\textrm{N}}_{(i-1)}$ DD–$\textrm{N}_{(i)}\textrm{H}^{\textrm{N}}_{(i)}$ DD), Experiment 4 (${\textrm{H}^\textrm{N}_{(i)}{\textrm{H}^{\alpha }_{(i-1)}}} DD-{\textrm{C}'_{(i-1)}} CSA$), and Experiment 5 (${\textrm{C}^\alpha _{(i-1)}} {\textrm{H}^\alpha _{(i-1)}}$ DD–${\textrm{C}'_{(i-1)}}$ CSA)) provide CCR rates that yield information on the dihedral angles of a residue preceding the detected residue, which gives us information about proline residues. In our analysis, we ignore the glycine residues due to the presence of two alpha protons, which makes it impossible to differentiate these during the experiment. In total, we obtained 452 CCR rates (83 % of all expected). We did not obtain 92 CCR rates due to the low intensity of the peaks in the reference version of the experiments. For 41 amino acid residues, we obtained the whole set of eight rates. We did not observe any correlation between the number of missing rates with amino acid type or secondary structure. The number of CCR rates obtained from the experiments is as follows: CCR 1: 55, CCR 2: 58, CCR 3: 53, CCR 4: 54, CCR 5: 60, CCR 6: 60, CCR 7: 53, and CCR 8: 59. The CCR rates are gathered in the Supplementary Information Tables 1 and 2.

**Fig. 11:**
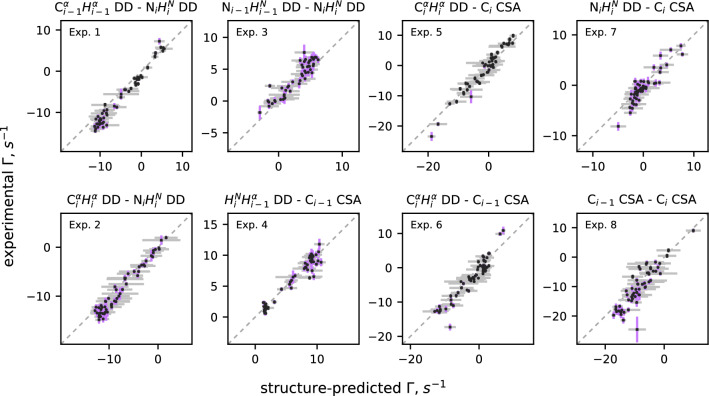
Comparison of experimental CCR rates with structure-predicted CCR rates based on 6v5d PDB structure. Pink vertical error bars, for experimental CCR rates, correspond to experimental uncertainty originating from the spectral noise. Grey horizontal lines reflect the variability of conformers submitted to PDB within the entry

To validate our techniques, we first compared the values of the experimentally obtained CCR rates with structure-predicted CCR rates calculated for the structure of ubiquitin deposited in PDB under the code 6V5D. Fig. 11 shows the comparison between the rates that we measured and the structure-predicted rates. The data are in very good agreement; the points align along the y = x line, with some scatter. A possible explanation for the scatter, besides the experimental uncertainty, is the variability of the structure-predicted rates (represented in the figure as grey horizontal lines), originating from the differences between the 176 deposited conformers.

**Fig. 12:**
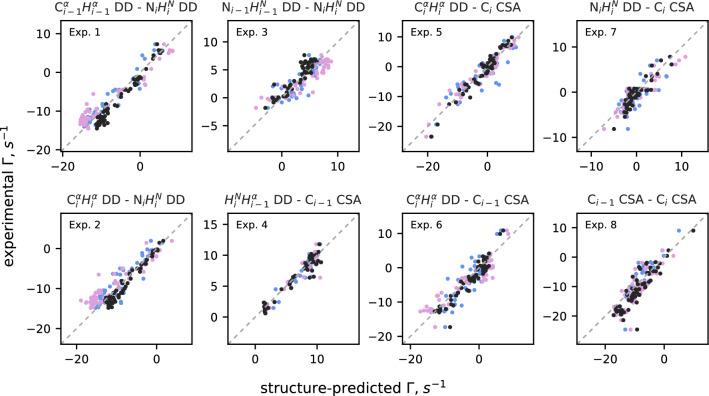
Comparison of experimental CCR rates with structure-predicted CCR rates based on three different PDB structures: 6V5D (black dots), 1D3Z (pink dots), and 2NR2 (blue dots)

The analogous comparisons for two different PDB structures (1D3Z and 2NR2) are shown in the Supplementary Information Figs. 1 and 2. The results are similar, however there are some differences in the slope for the individual structures (especially in Experiment 1 ($\textrm{C}^{\alpha }_{(i-1)}\textrm{H}^{\alpha }_{(i-1)}$ DD–$\textrm{N}_{(i)}\textrm{H}^{\textrm{N}}_{(i)}$ DD) and Experiment 2 ($\textrm{C}^{\alpha }_{(i)}\textrm{H}^{\alpha }_{(i)}$ DD–$\textrm{N}_{(i)}\textrm{H}^{\textrm{N}}_{(i)}$ DD)), which is clearly visible in Fig. 12, where all three comparisons are shown in a single chart. We suggest that this deviation may be due to the differences in the methods used for establishing the constraints for structure determination (NOE and RDC), which average differently over the structural ensemble. Notably, the CCR rate averaging is also different from the NOE and RDC effects averaging, which may influence the consistency of the results. What is remarkable is that also the slopes of the dependencies of the back-calculated CCR rates of two different PDB structures often deviate from 1 (see Supplementary Information Figs. 3, 4 and 5).

It is known that CCR experiments can greatly benefit from the principle of symmetrical reconversion (Pelupessy et al. 2003a). The experiments presented here may also be adapted to use this approach, which is expected to result in higher accuracy of the results. This would however require measurements of four versions of each experiments (two reference and two transfer) instead of two, thus doubling the measurement time.

**Fig. 13:**
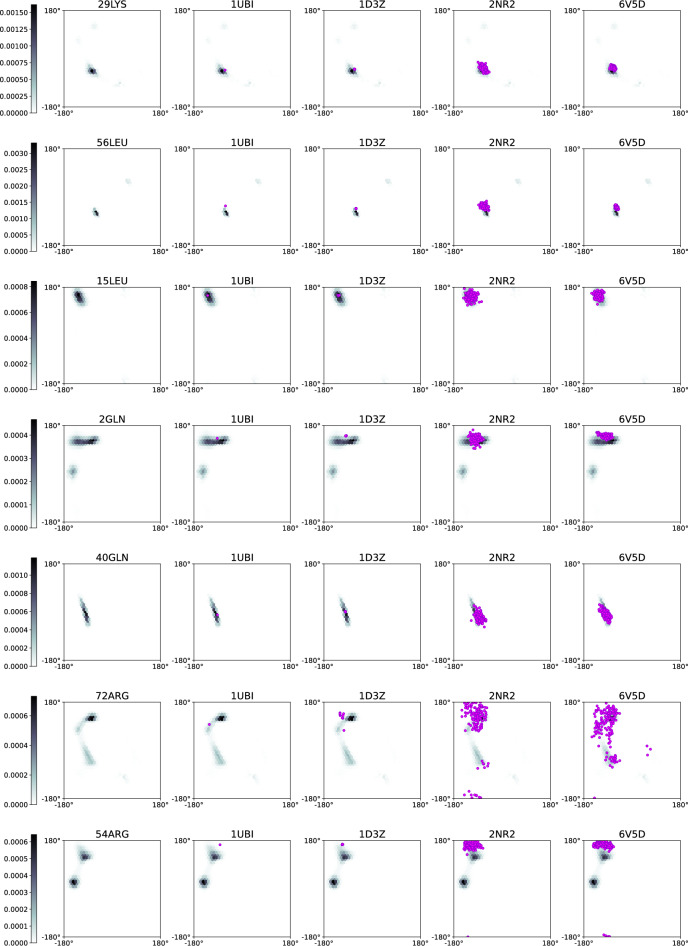
Backbone dihedral angle distribution plots for the following residues: 29Lys, 56Leu, 15Leu, 2Gln, 40Gln, 72Arg, and 54Arg. $\phi$ angles are shown on the x-axes and $\psi$ angles—on the y-axes. Each row represents a single residue. Figures in the first column show the probability densities of individual backbone conformations, as obtained from maximum entropy analysis. In columns 2–5, the maps are overlaid with the values of the backbone dihedral angles obtained from the ubiquitin structures deposited in the PDB database: 1UBI, 1D3Z, 2NR2, 6V5D

To assess the potential applicability of our approach to obtain meaningful $\phi$, $\psi$ distributions, we compared the distributions obtained using the maximum entropy approach (Kauffmann et al. 2021) with the available PDB data. Figure 13 shows the probability density of the backbone dihedral angles for seven selected residues involved in different structural motifs: 29Lys, 56Leu, 15Leu, 2Gln, 40Gln, 72Arg, and 54Arg. The plots were also overlaid with the values of the $\phi$ and $\psi$ angles obtained from the four PDB-deposited ubiquitin structures (crystallographic 1UBQ, as well as NMR 1D3Z, 2NR2, and 6V5D). A separate point is shown for each of the deposited conformers. Most of the distributions obtained are consistent with the structural data. For residues in helical structures (e.g. 29Lys, 56Leu), the agreement is excellent, as well as for the residues in the $\beta$-strand in the rigid fragment of the polypeptide chain (e.g. 15Leu). For more mobile $\beta$-strand residues (e.g. near the N- or C-terminus of the protein) we observe more spread distribution, although its maximum overlays with the PDB-derived $\phi$ and $\psi$ positions (e.g. 2Gln). The agreement is typically very good also for the residues involved in other types of structural elements, like turns (e.g. 40Gln), or disordered fragments of the polypeptide chain (e.g. 72Arg located near the C-terminus). We observe certain discrepancies between our distributions and PDB structures for only few residues (e.g. 54Arg). The ($\phi , \psi$) probability densities for all residues obtained through experiment can be found in Supplementary Information Fig. 6.

The distributions obtained from the CCR rates, utilising the maximum entropy method, reflect the structural heterogeneity of individual residues in ubiquitin. This heterogeneity is to a certain extent also covered by structural ensembles of 1D3Z, 2NR2 and 6V5D. The consistency of the data presented herein with those based on different NMR parameters, which are observed for more dynamic residues, suggests that there are favourable prospects for future studies of disordered regions of polypeptide chains.

## Conclusion

We present a comprehensive set of eight 4D experiments for measuring CCR rates. We validated the experiments using a ubiquitin sample and PDB-deposited structural data for this protein. The rates, analyzed using a maximum entropy approach, provide the distributions of backbone dihedral angles for each individual amino acid residue. The distributions for the majority of residues are consistent with respective $\phi$, $\psi$ values derived from PDB data. Their maxima correspond to the typical $\phi$, $\psi$ values of a given secondary structure. At the same time, they reflect the mobility of the polypeptide chain, which will be particularly valuable when dealing with intrinsically disordered proteins, as well as long and flexible fragments of mostly folded proteins: loops, interdomain linkers, N- and C-termini. Using this method with such proteins opens up a new way to determine structural ensembles.

## Supplementary Information

Below is the link to the electronic supplementary material.

Supplementary file 1 (pdf 12461 KB)

## Data Availability

Data is provided within the manuscript and supplementary information files.

## Code availability

All the pulse sequences and both scripts for data analysis (automatic adjustment of peaks positions for the measured spectra and calculation of the CCR rates) are deposited on GitHub: https://github.com/nmr-cnbch/CCR_pulse_sequences_and_analysis_scripts/releases/latest.
