# Supplementary material for: A complete set of cross-correlated relaxation experiments for determining the protein backbone dihedral angles
Source: J Biomol NMR. 2025 Mar 20;79(2):79–98. doi: 10.1007/s10858-025-00458-x (PMC12078423; doi:10.1007/s10858-025-00458-x)
Supplement: Supplementary file 1 — (pdf 12461 KB) [file 10858_2025_458_MOESM1_ESM.pdf]

Supplementary Information to article: "A complete set of cross-correlated relaxation experiments for protein backbone dihedral angle determination"

Paulina Bartosińska-Marzec[1], Bartłomiej Banaś[1], Clemens Kauffmann[2,3], Andreas Beier[2], Daniel Braun[2], Irene Ceccolini[2], Wiktor Koźmiński[1], Robert Konrat[2], Anna Zawadzka-Kazimierczuk[1]

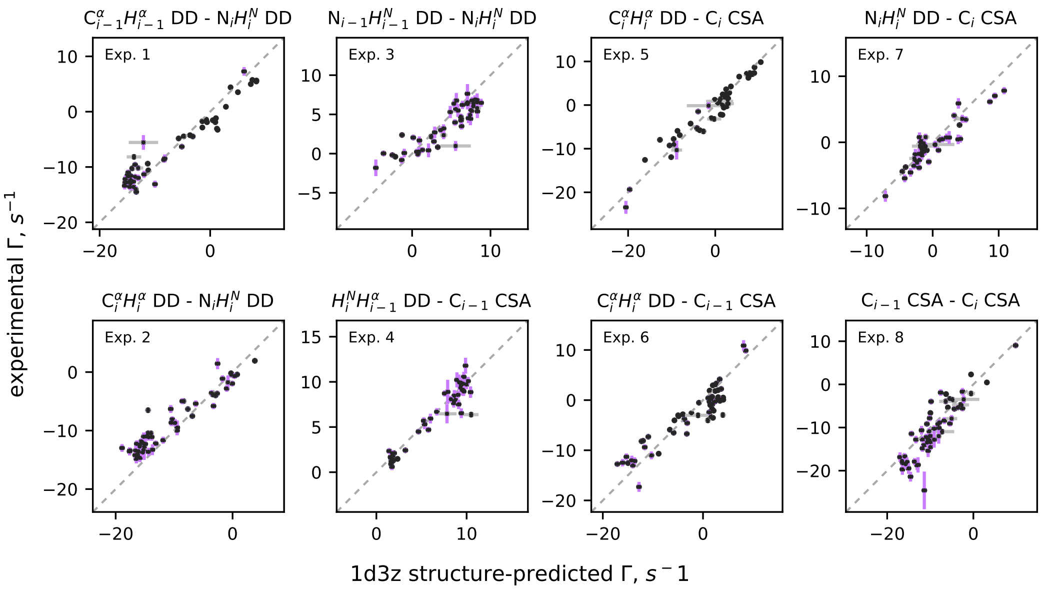

Fig. 1 The comparison of experimental CCR rates with structure-predicted CCR rates based on 1D3Z PDB structure. Pink vertical error bars, for experimental CCR rates, correspond to experimental uncertainty originating from the spectral noise. Grey horizontal lines reflect the variability of conformers submitted to PDB within the entry.

[1] Biological and Chemical Research Centre, Faculty of Chemistry, University of Warsaw, Żwirki i Wigury 101, 02-089 Warsaw, Poland

[2] Department of Structural and Computational Biology, Max Perutz Laboratories, University of Vienna, Vienna Biocenter Campus 5, A-1030 Vienna, Austria

[3] Wiener Linien GmbH & Co KG, Erdbergstraße 202, 1030 Vienna, Austria

E-mail: Robert Konrat robert.konrat@univie.ac.at

E-mail: Anna Zawadzka-Kazimierczuk anzaw@chem.uw.edu.pl

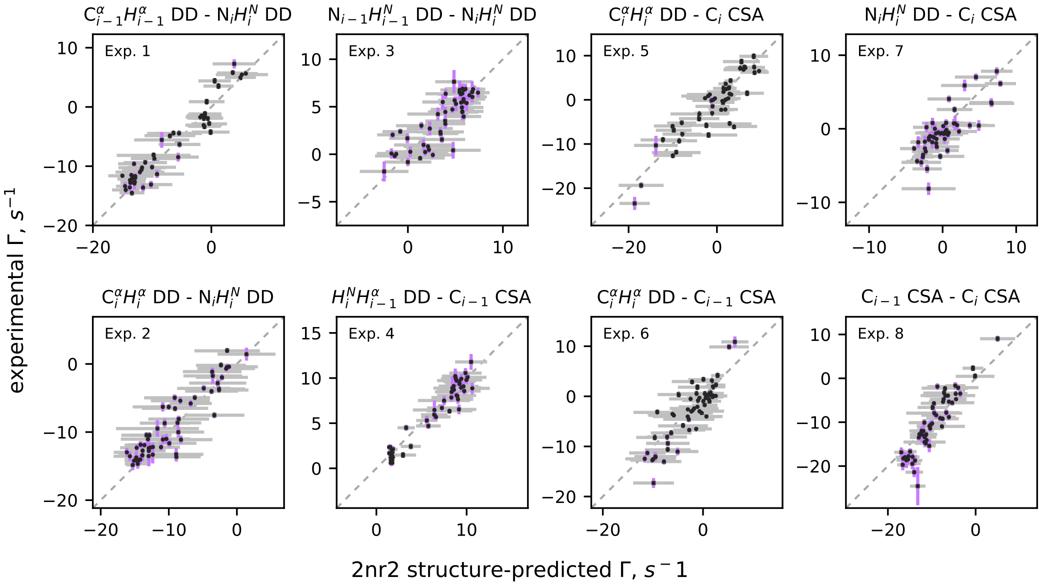

Fig. 2 The comparison of experimental CCR rates with structure-predicted CCR rates based on 2NR2 PDB structure. Pink vertical error bars, for experimental CCR rates, correspond to experimental uncertainty originating from the spectral noise. Grey horizontal lines reflect the variability of conformers submitted to PDB within the entry.

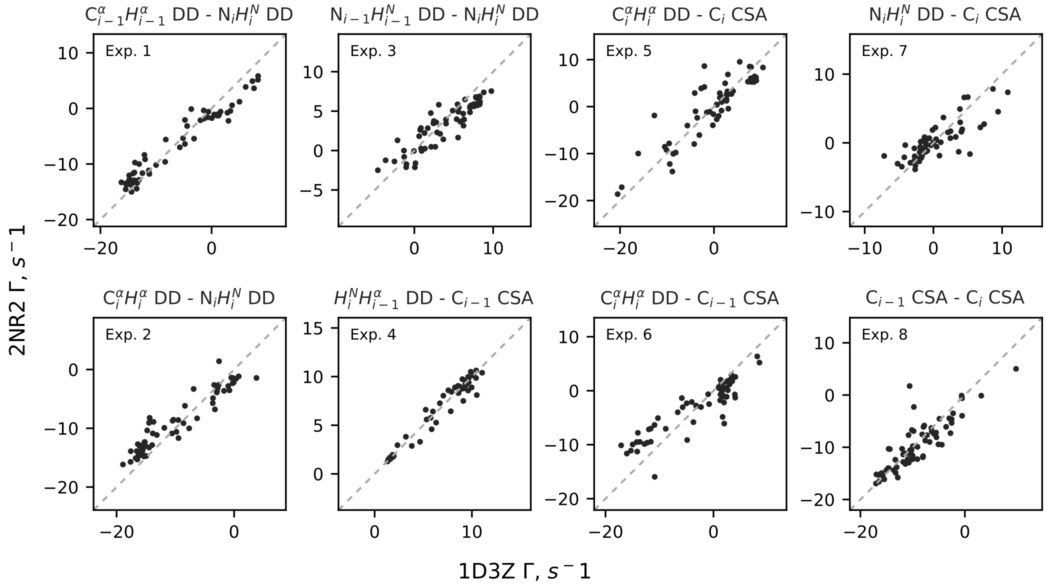

Fig. 3 The comparison of structure-predicted CCR rates based on 1D3Z PDB structures and 2NR2 PDB structures

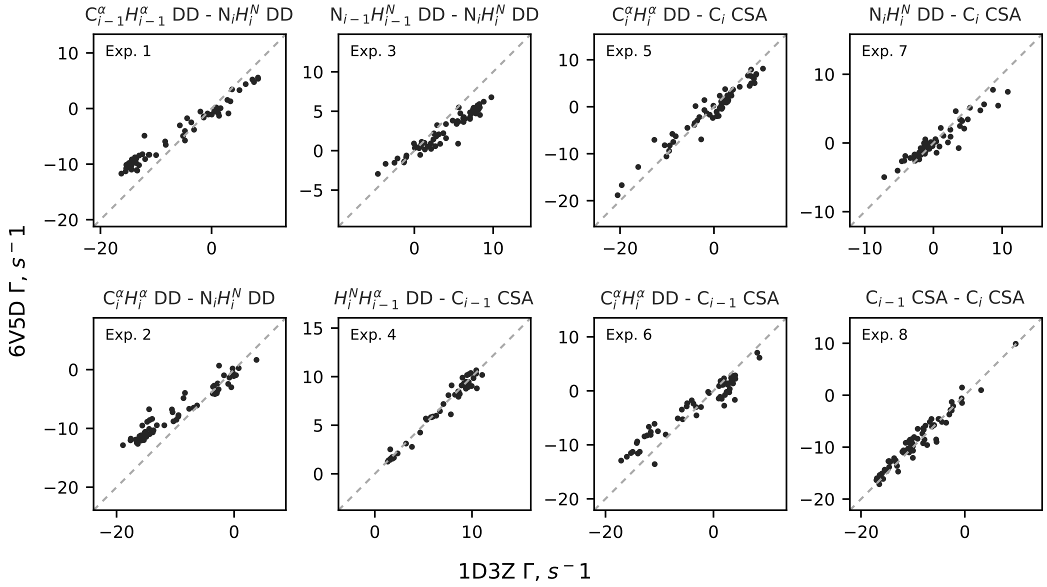

Fig. 4 The comparison of structure-predicted CCR rates based on 1D3Z PDB structures and 6V5D PDB structures

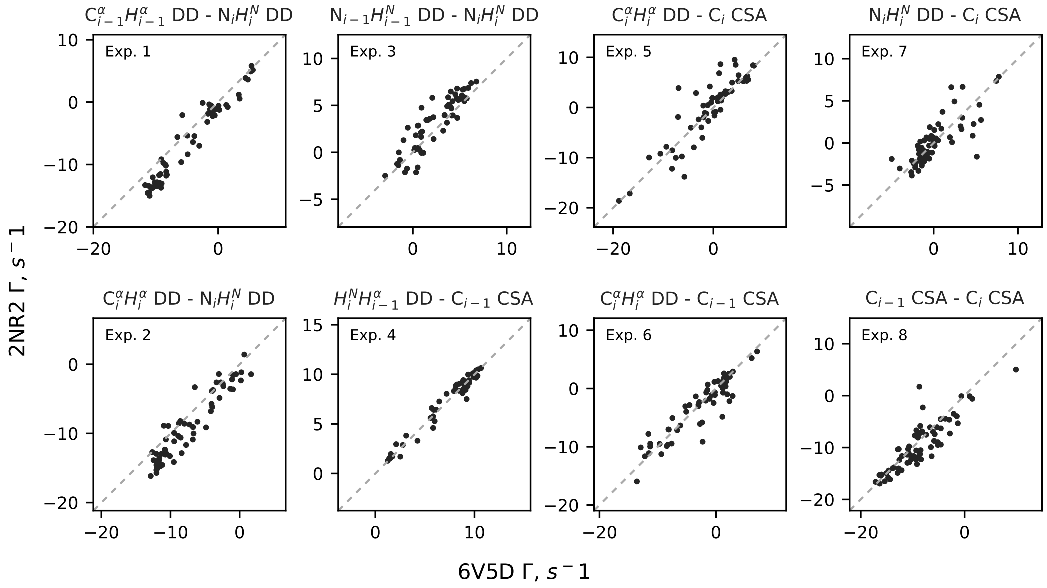

Fig. 5 The comparison of structure-predicted CCR rates based on 6V5D PDB structures and 2NR2 PDB structures

Table 1 CCR rates values and standard deviation for experiments no. 1-4

res	1	st. dev.	2	st. dev.	3	st. dev.	4	st. dev.
1MET	-8.198	0.222	nan	nan	nan	nan	5.5777	0.2355
2GLN	-11.6159	0.3779	-10.4662	0.3529	1.5517	0.3544	9.7956	0.4462
3ILE	-4.4788	0.3657	-11.8627	0.5454	6.7685	0.9655	5.6973	0.3751
4PHE	-11.7767	0.4855	-14.8282	0.7792	5.3842	0.8519	7.6423	0.4882
5VAL	-12.7816	0.4835	-14.1756	0.7307	6.8665	0.5401	9.0833	0.5703
6LYS	-14.0029	0.5766	-12.7306	0.813	3.2072	0.5282	8.5618	0.4014
7THR	nan	nan	-11.0069	0.7181	nan	nan	nan	nan
8LEU	nan	nan	-1.7535	1.1709	nan	nan	nan	nan
9THR	nan	nan	nan	nan	nan	nan	nan	nan
10GLY	nan	nan	nan	nan	nan	nan	nan	nan
11LYS	nan	nan	-6.306	0.6746	nan	nan	nan	nan
12THR	-12.6408	0.7393	-12.7614	2.2568	7.6314	1.2338	10.2158	0.8363
13ILE	-9.5972	0.437	-13.533	0.8867	3.4949	0.4321	8.0142	0.4239
14THR	-13.5844	0.6279	-12.1991	0.6458	6.3658	0.5161	9.355	0.5277
15LEU	-10.6008	0.3167	-14.204	0.6272	4.9276	0.4999	8.7164	0.344
16GLU	-12.8038	0.4793	-11.9147	0.4189	4.7124	0.3734	10.55	0.5598
17VAL	-4.4685	0.2641	-12.3038	0.554	6.3192	0.6858	4.6959	0.3184
18GLU	nan	nan	nan	nan	nan	nan	nan	nan
19PRO	-0.2829	0.2012	nan	nan	-0.1248	0.7396	1.2909	0.2234
20SER	4.9868	0.2482	-10.0033	0.8017	2.3762	0.2676	1.3626	0.1965
21ASP	-10.2517	0.3596	-5.4025	0.561	-0.1753	0.3012	9.7181	0.5344
22THR	-8.4672	0.7429	-8.648	0.5891	0.0787	0.5412	nan	nan
23ILE	nan	nan	-1.127	0.5716	nan	nan	nan	nan
24GLU	nan	nan	nan	nan	nan	nan	nan	nan
25ASN	-1.5491	0.2364	nan	nan	6.3983	0.3899	1.5609	0.287
26VAL	-1.7286	0.2184	-3.6644	0.3978	6.8449	0.4344	1.2153	0.3078
27LYS	-3.0725	0.2939	-1.9609	0.4619	nan	nan	2.1002	0.3443
28ALA	-1.1775	0.221	-2.8091	0.5585	6.7706	0.4562	1.4334	0.2212
29LYS	-1.8961	0.3161	-3.9917	0.3598	4.4407	0.819	1.6863	0.3643
30ILE	-1.8934	0.2494	-3.5872	0.4822	5.5441	0.7816	0.9034	0.289
31GLN	-3.2702	0.2474	-0.6763	0.4796	6.4788	0.5144	1.2745	0.1935
32ASP	-1.451	0.1966	-0.411	0.3432	6.0315	0.2817	1.4612	0.197
33LYS	-1.4481	0.2727	-7.5255	0.3732	6.2081	0.5202	2.3406	0.3197
34GLU	4.4006	0.3188	-11.0945	0.6418	-0.0747	0.3445	1.6783	0.3677
35GLY	nan	nan	nan	nan	nan	nan	nan	nan
36ILE	nan	nan	-9.4911	1.2138	nan	nan	nan	nan
37PRO	nan	nan	nan	nan	nan	nan	nan	nan
38PRO	-1.9943	0.2119	nan	nan	-0.1631	0.82	1.2838	0.2058
39ASP	3.5224	0.1973	-3.8281	0.3356	2.1776	0.2459	1.5873	0.2214
40GLN	5.734	0.2543	-13.6275	0.6913	-0.3956	0.2202	1.7843	0.2178
41GLN	-14.4989	0.4057	-11.1153	0.5929	3.004	0.3972	9.6042	0.5515
42ARG	-12.1886	0.6754	-14.6626	0.7136	6.5513	1.1257	9.111	0.6655
43LEU	-12.7039	0.5494	-13.7376	0.825	5.3047	0.7804	8.8642	0.6365
44ILE	-11.9724	0.6045	-14.2246	0.7072	5.448	0.7801	9.9484	0.8601
45PHE	nan	nan	-11.0449	0.7182	nan	nan	nan	nan
46ALA	nan	nan	nan	nan	nan	nan	3.2123	0.5167
47GLY	nan	nan	nan	nan	nan	nan	nan	nan
48LYS	-10.9988	0.4879	-13.4114	0.4995	4.2294	0.6904	8.9396	0.4994
49GLN	-13.5961	0.6582	-6.3264	0.3757	0.2306	0.3951	8.4692	0.6286
50LEU	-11.6853	0.4959	-8.0897	0.4949	0.2579	0.553	6.527	0.5386
51GLU	-13.1061	0.6404	-11.646	0.6015	nan	nan	8.8867	1.3417
52ASP	nan	nan	1.4401	0.9508	nan	nan	nan	nan
53GLY	nan	nan	nan	nan	nan	nan	nan	nan
54ARG	-4.8359	0.3361	-13.5547	0.6978	0.4179	0.8812	5.9342	0.5409
55THR	-6.3269	0.4281	-13.324	1.2857	1.6775	0.5151	5.3043	0.5445
56LEU	-2.8607	0.2052	-0.2043	0.3052	6.8945	0.5319	1.6087	0.2957
57SER	nan	nan	-0.4515	0.4819	5.6701	0.3751	nan	nan
58ASP	0.8894	0.2628	nan	nan	3.9726	0.4148	0.5962	0.3625
59TYR	5.6333	0.2766	-13.2759	0.9215	0.0237	0.3308	2.1047	0.2463
60ASN	7.2784	0.7851	nan	nan	4.5124	0.623	2.4258	0.2662
61ILE	-11.4998	0.3971	-8.6896	0.6501	0.472	0.3637	10.0831	0.4691
62GLN	-4.2488	0.2419	-12.2204	0.4936	2.0441	0.3474	4.4945	0.2768
63LYS	-11.3306	0.6609	1.9289	0.3184	-1.8116	1.0588	7.514	0.5255
64GLU	5.4616	0.2529	-5.8001	0.5319	0.8263	0.2715	1.4714	0.172

res	1	st. dev.	2	st. dev.	3	st. dev.	4	st. dev.
65SER	-9.3946	0.3473	-4.9685	0.5002	-0.8213	0.4335	8.079	0.3735
66THR	-12.9187	0.9957	-12.3668	0.6833	4.4505	0.5587	nan	nan
67LEU	-8.7103	0.4163	-13.4229	0.8762	2.7099	0.7315	6.6849	0.4063
68HIS	-12.3657	0.3488	-14.0298	0.6665	6.5317	0.5751	9.9169	0.5583
69LEU	-13.3838	0.6753	-12.2591	0.5936	5.497	0.8221	11.7905	0.8764
70VAL	-8.1722	0.2049	-12.9733	0.664	5.8015	0.2881	6.3734	0.199
71LEU	-10.1822	0.4417	-10.4754	0.354	2.3494	0.508	7.8455	0.4275
72ARG	-5.5618	1.3309	-6.516	0.3218	0.9772	0.6384	6.4681	1.0582
73LEU	nan	nan	-4.969	0.3795	nan	nan	nan	nan
74ARG	nan	nan	nan	nan	nan	nan	nan	nan
75GLY	nan	nan	nan	nan	nan	nan	nan	nan
76GLY	nan	nan	nan	nan	nan	nan	nan	nan

Table 2 CCR rates values and standard deviation for experiments no. 5-8

res	5	st. dev.	6	st. dev.	7	st. dev.	8	st. dev.
1MET	-5.4302	0.2293	nan	nan	nan	nan	nan	nan
2GLN	0.1089	0.1589	-2.6106	0.2773	-3.0775	0.4986	-6.6318	0.38
3ILE	-9.2651	0.4444	0.1965	0.3107	0.7272	0.6042	0.4535	0.3732
4PHE	-2.2503	0.2374	0.6864	0.4065	0.0038	0.6809	-5.3256	0.5339
5VAL	2.8223	0.2437	-0.1401	0.3374	-1.4742	0.4112	-11.9666	0.8775
6LYS	1.3729	0.1877	0.3989	0.4166	-1.7541	0.8427	-10.8712	0.7713
7THR	-10.3183	2.1586	-2.1038	0.2846	3.5574	0.6084	-3.5415	0.4653
8LEU	nan	nan	-11.0163	0.7762	nan	nan	-11.9935	1.7443
9THR	-8.7725	0.6865	nan	nan	nan	nan	nan	nan
10GLY	nan	nan	nan	nan	nan	nan	nan	nan
11LYS	nan	nan	-4.5831	0.5125	-1.8233	0.8217	-3.9734	0.586
12THR	3.6188	0.2895	0.1782	0.5405	nan	nan	-12.8237	1.8441
13ILE	-2.1787	0.2289	3.3206	0.3828	0.2036	0.6817	-10.1051	0.8156
14THR	2.4527	0.2476	-0.9349	0.2984	-3.8364	0.4401	-12.233	0.7858
15LEU	-3.0137	0.1735	3.1446	0.3415	-0.656	0.5077	-2.1151	0.4476
16GLU	4.2665	0.2713	-0.9796	0.2857	-3.545	0.5453	nan	nan
17VAL	-5.9192	0.2352	1.8954	0.329	-0.3662	0.4397	9.0282	0.5401
18GLU	nan	nan	1.9812	0.3036	nan	nan	-3.3536	0.4238
19PRO	3.1853	0.1516	nan	nan	nan	nan	nan	nan
20SER	-11.9412	0.4171	-2.8256	0.2679	6.1384	0.4415	-14.0888	0.9237
21ASP	0.3699	0.2306	-9.3936	0.5634	-3.7497	0.3142	-10.6371	1.0118
22THR	-5.2545	0.4966	-6.7249	0.4596	0.4751	0.488	-8.8404	0.7437
23ILE	nan	nan	-12.1438	0.7357	nan	nan	-18.2595	1.5255
24GLU	7.2504	0.4067	nan	nan	nan	nan	nan	nan
25ASN	7.0869	0.2778	nan	nan	-0.8092	0.527	nan	nan
26VAL	7.3351	0.2223	-8.2057	0.4116	-0.6323	0.3251	-19.7028	1.2584
27LYS	8.6578	0.3061	-11.3014	0.6626	-1.3776	0.6378	-19.4722	1.2524
28ALA	6.8233	0.2419	-12.0236	0.946	-0.5348	0.3734	-17.7516	1.0232
29LYS	7.2416	0.2613	-8.1046	0.4137	-0.1594	0.5421	-16.7085	0.7528
30ILE	7.1864	0.2127	-7.3076	0.4386	-2.955	0.4935	-18.4954	1.1264
31GLN	9.8534	0.2534	-12.4765	0.7441	-1.9705	0.3574	-16.8624	1.1156
32ASP	6.245	0.1763	-12.5527	0.3827	-1.2016	0.2525	-17.9841	1.1037
33LYS	6.5196	0.1922	-3.2072	0.2025	-1.2024	0.4105	-17.9025	0.8326
34GLU	-6.0282	0.2274	0.5086	0.2894	4.0538	0.4696	-5.6849	0.5409
35GLY	nan	nan	nan	nan	nan	nan	nan	nan
36ILE	nan	nan	-4.0348	0.3058	nan	nan	-11.4428	0.6915
37PRO	nan	nan	nan	nan	nan	nan	nan	nan
38PRO	7.1736	0.2106	nan	nan	nan	nan	nan	nan
39ASP	-5.4472	0.1832	-10.6824	0.326	2.6177	0.3114	-12.7492	0.7146
40GLN	-7.9734	0.2145	0.3552	0.233	7.0303	0.4856	-13.4673	0.7407
41GLN	2.6157	0.2095	-3.5278	0.3639	-2.6974	0.3102	-9.08	0.6735
42ARG	-0.2708	0.2859	2.2378	0.4324	-1.0931	0.8595	-14.5346	0.7459
43LEU	2.531	0.3068	-0.6414	0.4843	nan	nan	-10.2881	0.7864
44ILE	-0.5781	0.2425	4.1517	0.4202	0.7745	0.7209	-4.7863	0.5281
45PHE	nan	nan	-2.4295	0.482	nan	nan	-7.9374	0.828
46ALA	-16.9718	1.46	nan	nan	nan	nan	nan	nan
47GLY	nan	nan	nan	nan	nan	nan	nan	nan
48LYS	-0.0046	0.1756	1.8223	0.2499	-0.5038	0.4781	-2.2429	0.3767
49GLN	2.5932	0.2055	-6.5044	0.3831	-5.4542	0.5911	-14.8256	0.808
50LEU	1.8058	0.2064	-3.7997	0.4341	-4.5627	0.6349	-9.168	0.565
51GLU	-1.4827	0.553	-0.5162	0.3937	nan	nan	-1.8963	0.7695
52ASP	nan	nan	nan	nan	nan	nan	-24.5731	4.3702
53GLY	nan	nan	nan	nan	nan	nan	nan	nan
54ARG	-7.7685	0.3903	0.5913	0.3357	0.5095	0.5485	-5.5589	0.6473
55THR	-6.8931	0.4073	-0.3261	0.5058	0.4427	0.7255	-1.688	0.8451
56LEU	7.3415	0.2137	-13.0167	0.5335	-0.6958	0.5284	-21.3974	1.1225
57SER	nan	nan	-17.3043	0.9873	-0.3966	0.4773	-18.7344	1.1043
58ASP	1.491	0.2166	nan	nan	0.3627	0.3568	nan	nan
59TYR	-12.5763	0.4132	2.949	0.3513	7.8201	0.5427	-15.3851	1.4543
60ASN	-23.4508	1.474	10.8752	1.0881	-0.6421	0.749	-18.6634	1.7734
61ILE	0.3308	0.1966	-3.0224	0.3677	-4.4241	0.3208	-12.8002	0.8685
62GLN	-9.0717	0.2475	-1.7597	0.2485	3.4475	0.4974	2.3056	0.3262
63LYS	1.207	0.2185	-12.7468	0.4942	-8.1472	0.8516	-5.3516	0.3486
64GLU	-19.3633	0.5656	9.8689	0.5819	5.8859	0.7903	-12.7622	0.7183

res	5	st. dev.	6	st. dev.	7	st. dev.	8	st. dev.
65SER	-5.7685	0.2653	-5.8986	0.367	-1.3747	0.2555	-7.8343	0.7247
66THR	2.4138	0.3344	-0.3041	0.351	-2.6742	0.586	-12.9345	0.7478
67LEU	-4.2494	0.2397	0.5051	0.4148	0.7249	0.9677	-8.3971	0.8013
68HIS	-0.0581	0.1888	0.1536	0.298	-0.9949	0.5988	-9.2021	0.6302
69LEU	1.1928	0.245	-0.8253	0.4164	-1.8271	0.8144	-13.3879	0.7635
70VAL	-3.1863	0.1237	0.0836	0.2641	0.793	0.5035	-3.9334	0.5097
71LEU	0.3215	0.1781	-4.0338	0.2507	-2.4457	0.4732	nan	nan
72ARG	0.8544	0.2834	-2.9649	0.222	-0.5773	0.5468	-10.9526	0.4535
73LEU	-0.1344	0.7973	-3.3195	0.1511	-0.3251	0.5783	-4.7555	0.3837
74ARG	nan	nan	-3.0303	0.2932	nan	nan	-3.4827	0.9098
75GLY	nan	nan	nan	nan	nan	nan	nan	nan
76GLY	nan	nan	nan	nan	nan	nan	nan	nan

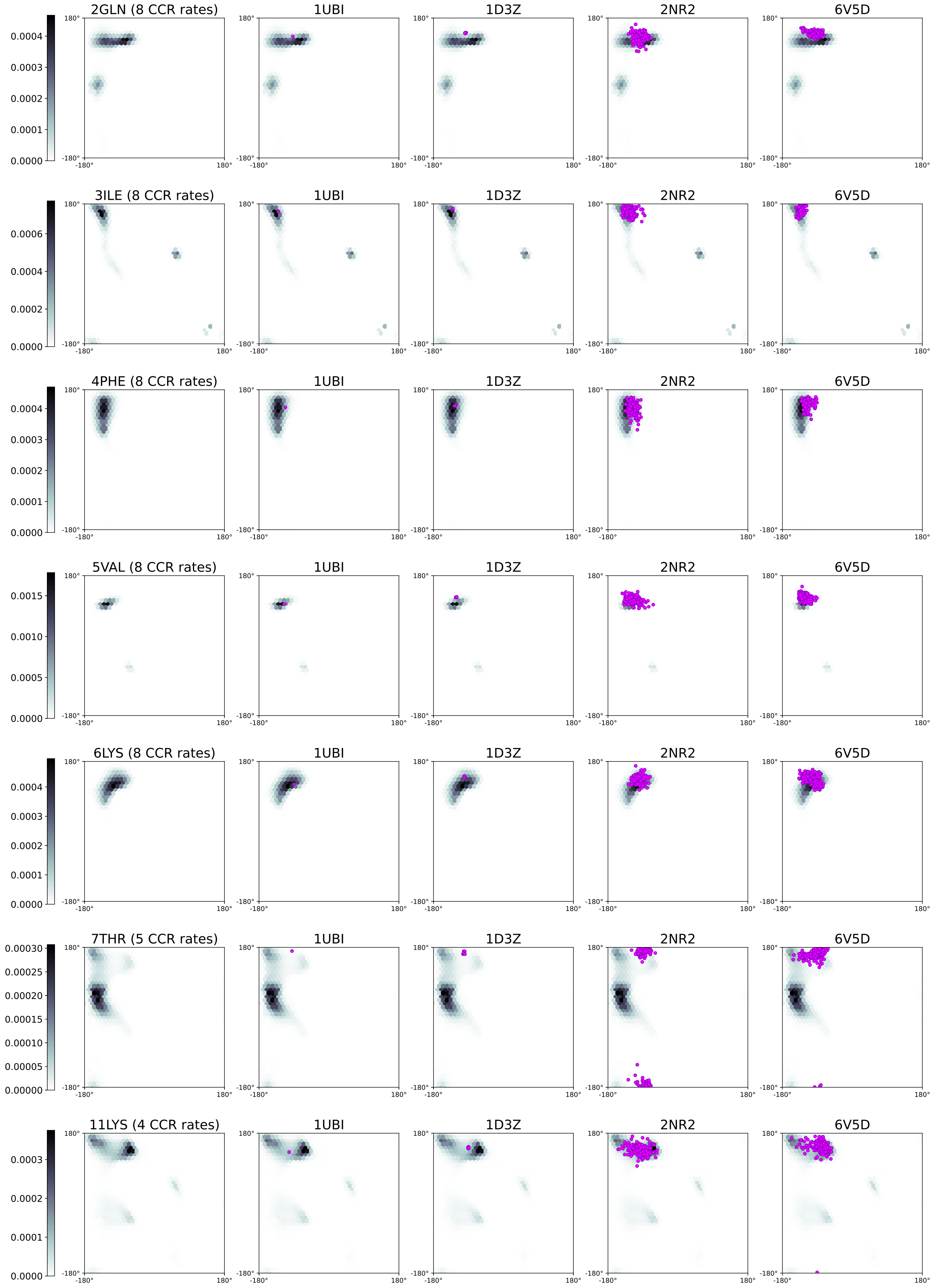
2GLN (8 CCR rates)
1UBI
1D3Z
2NR2
6V5D
3ILE (8 CCR rates)
4PHE (8 CCR rates)
5VAL (8 CCR rates)
6LYS (8 CCR rates)
7THR (5 CCR rates)
11LYS (4 CCR rates)
0.0000
0.0001
0.0002
0.0003
0.0004
0.0006
0.0005
0.0010
0.0015
0.00000
0.00005
0.00010
0.00015
0.00020
0.00025
0.00030
180°
-180°

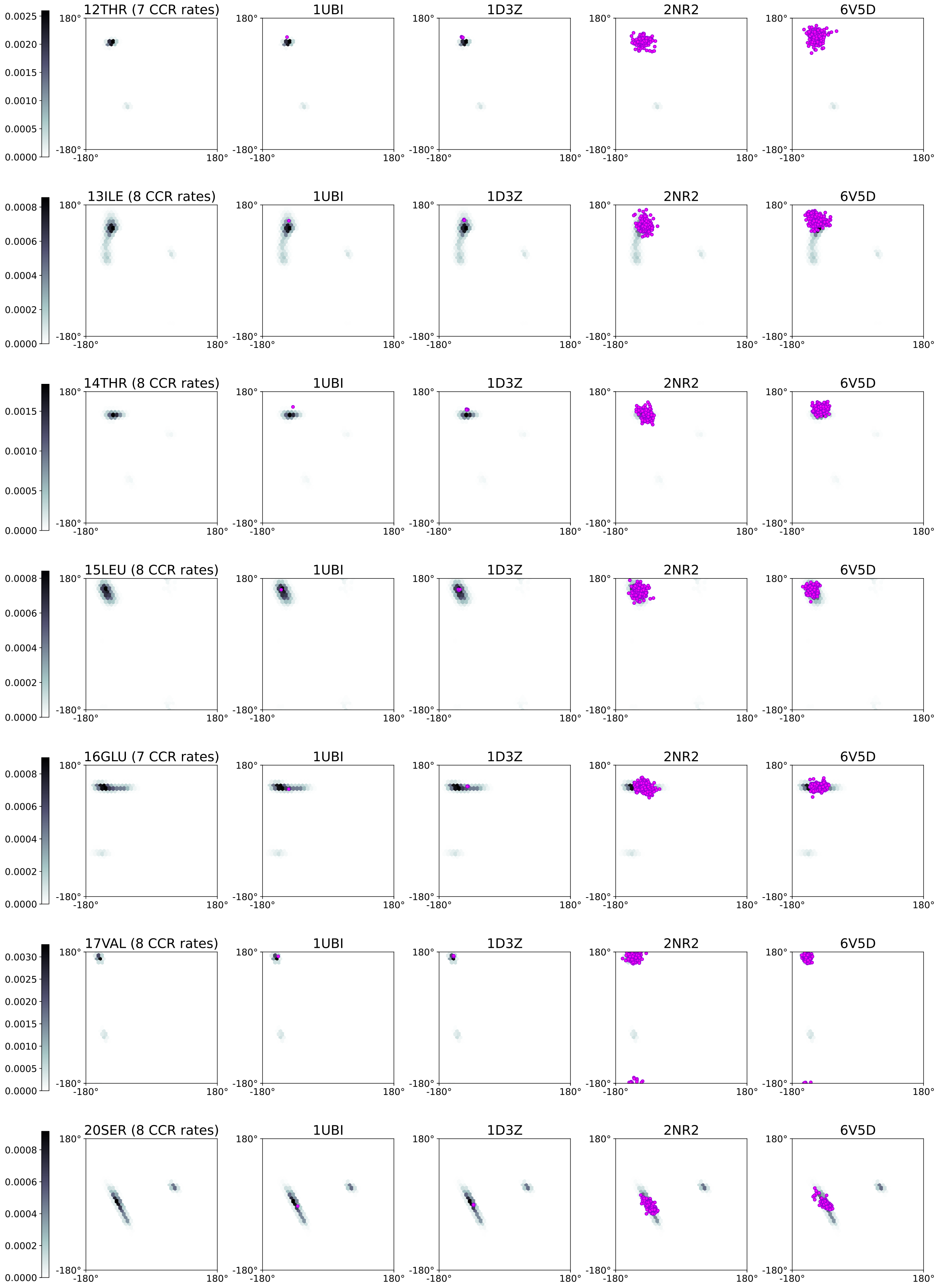

12THR (7 CCR rates)
13ILE (8 CCR rates)
14THR (8 CCR rates)
15LEU (8 CCR rates)
16GLU (7 CCR rates)
17VAL (8 CCR rates)
20SER (8 CCR rates)
1UBI
1D3Z
2NR2
6V5D
180°
-180°

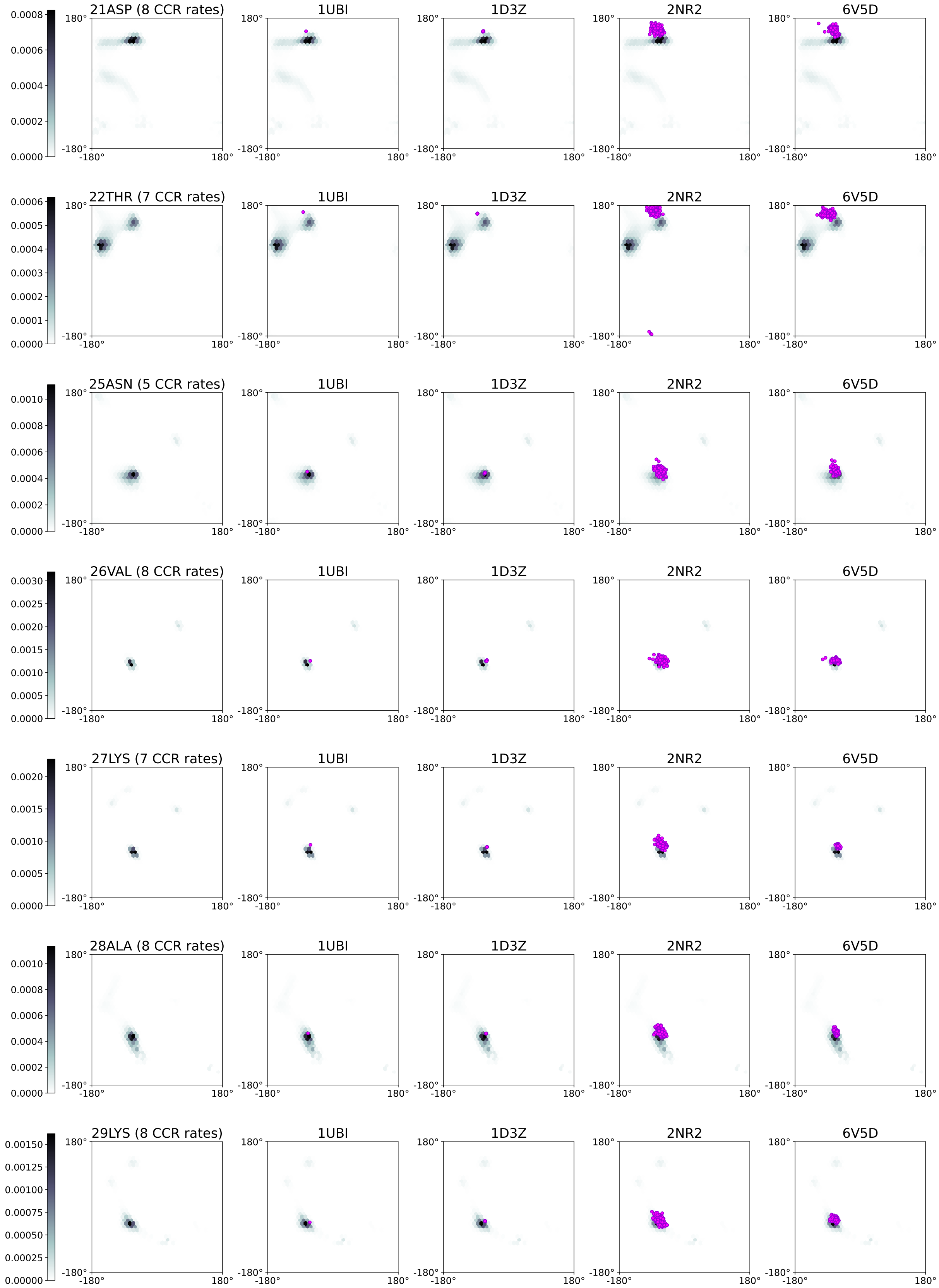
21ASP (8 CCR rates)
1UBI
1D3Z
2NR2
6V5D
22THR (7 CCR rates)
25ASN (5 CCR rates)
26VAL (8 CCR rates)
27LYS (7 CCR rates)
28ALA (8 CCR rates)
29LYS (8 CCR rates)
180°
-180°

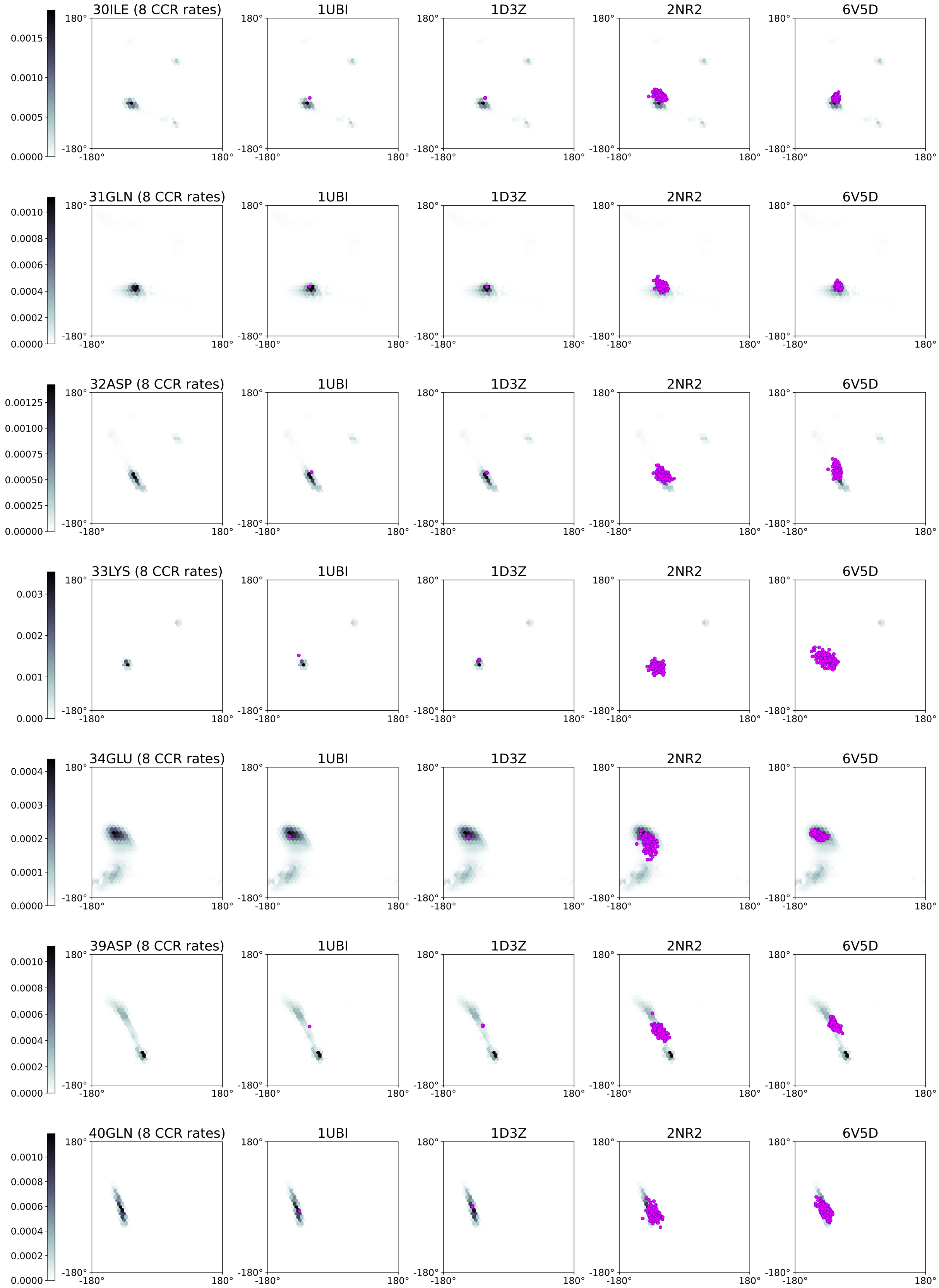
30ILE (8 CCR rates)
31GLN (8 CCR rates)
32ASP (8 CCR rates)
33LYS (8 CCR rates)
34GLU (8 CCR rates)
39ASP (8 CCR rates)
40GLN (8 CCR rates)
1UBI
1D3Z
2NR2
6V5D

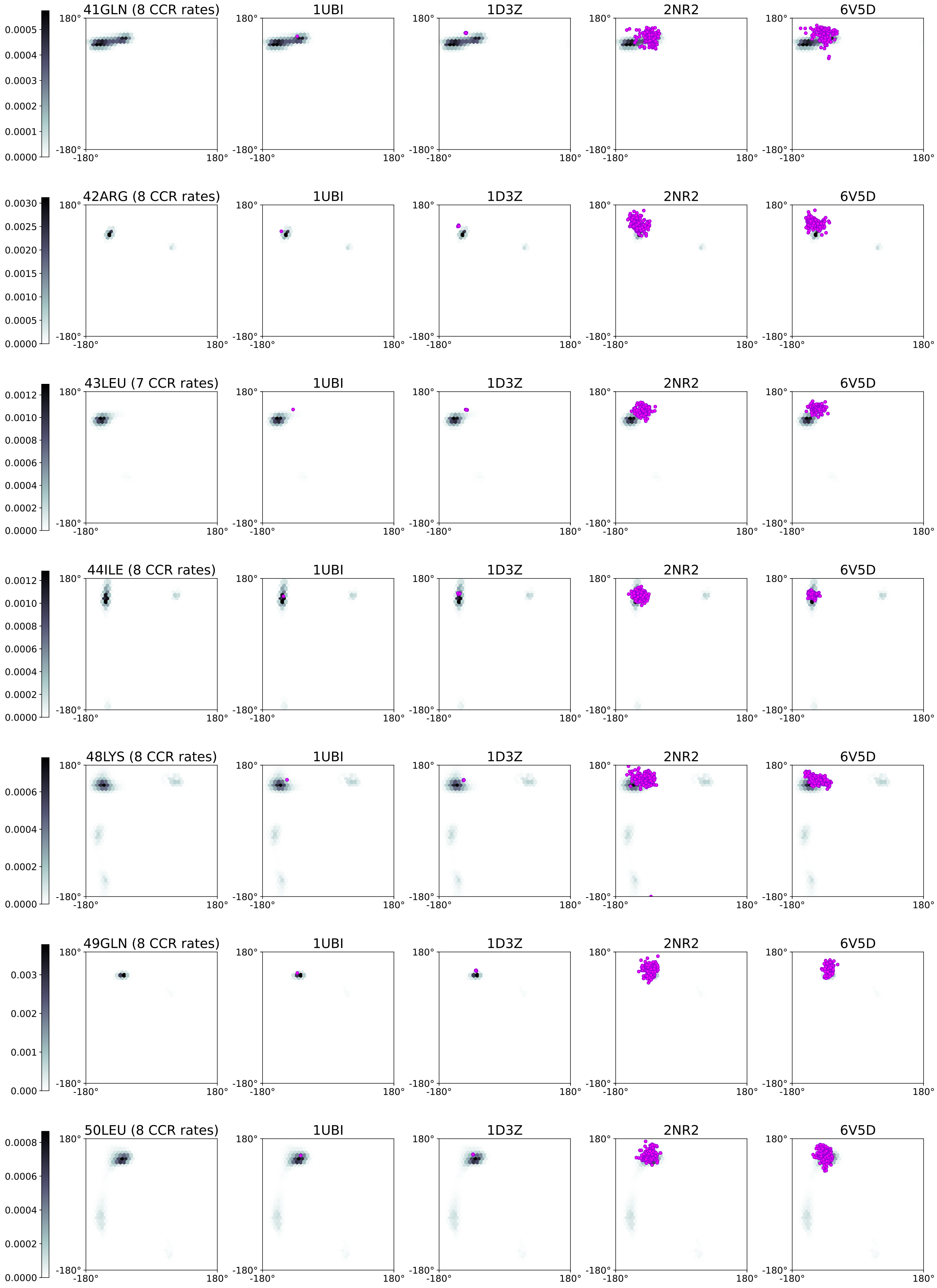

41GLN (8 CCR rates)
42ARG (8 CCR rates)
43LEU (7 CCR rates)
44ILE (8 CCR rates)
48LYS (8 CCR rates)
49GLN (8 CCR rates)
50LEU (8 CCR rates)
1UBI
1D3Z
2NR2
6V5D
180°
-180°

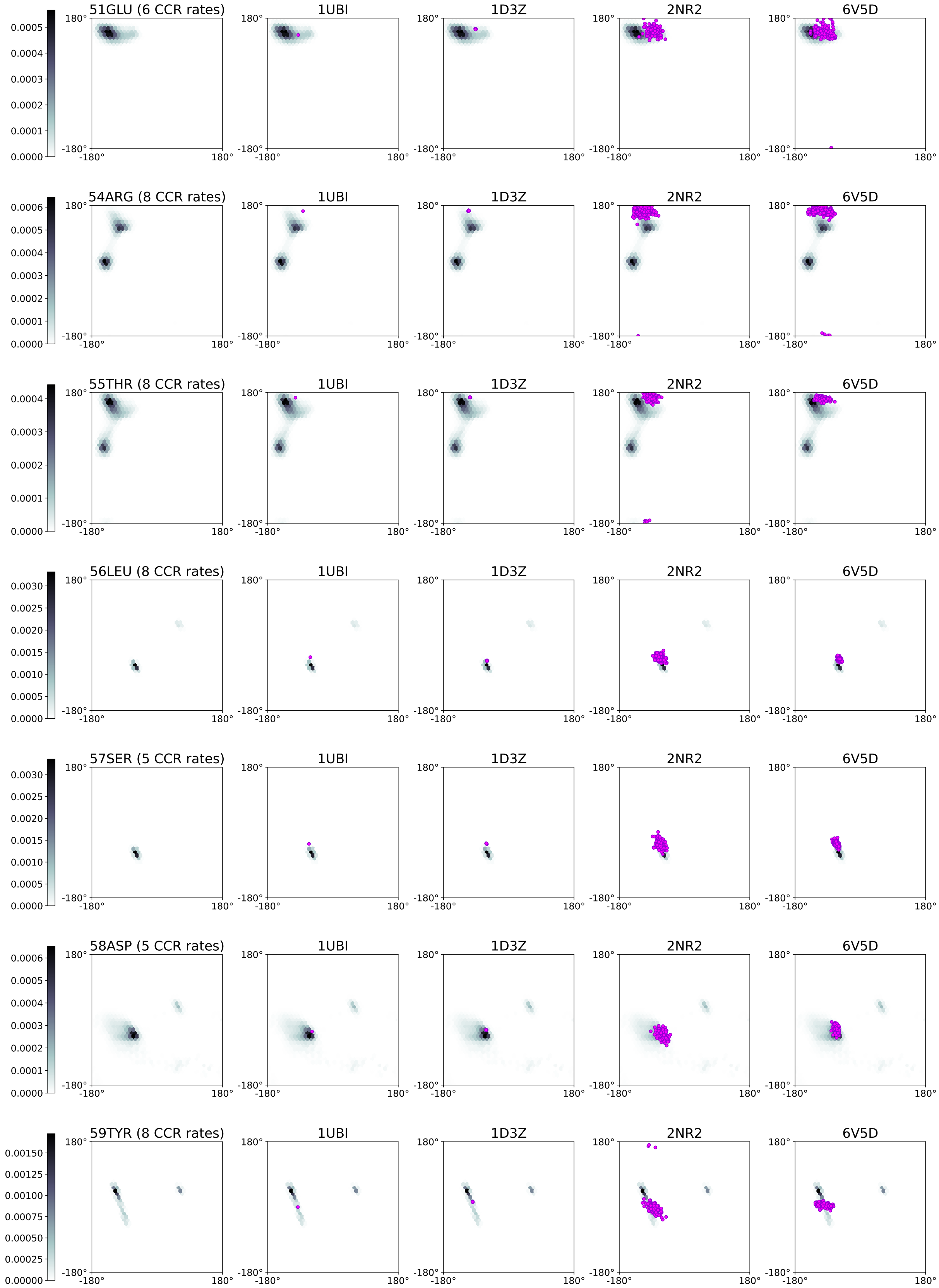
51GLU (6 CCR rates)
54ARG (8 CCR rates)
55THR (8 CCR rates)
56LEU (8 CCR rates)
57SER (5 CCR rates)
58ASP (5 CCR rates)
59TYR (8 CCR rates)
1UBI
1D3Z
2NR2
6V5D
180°
-180°

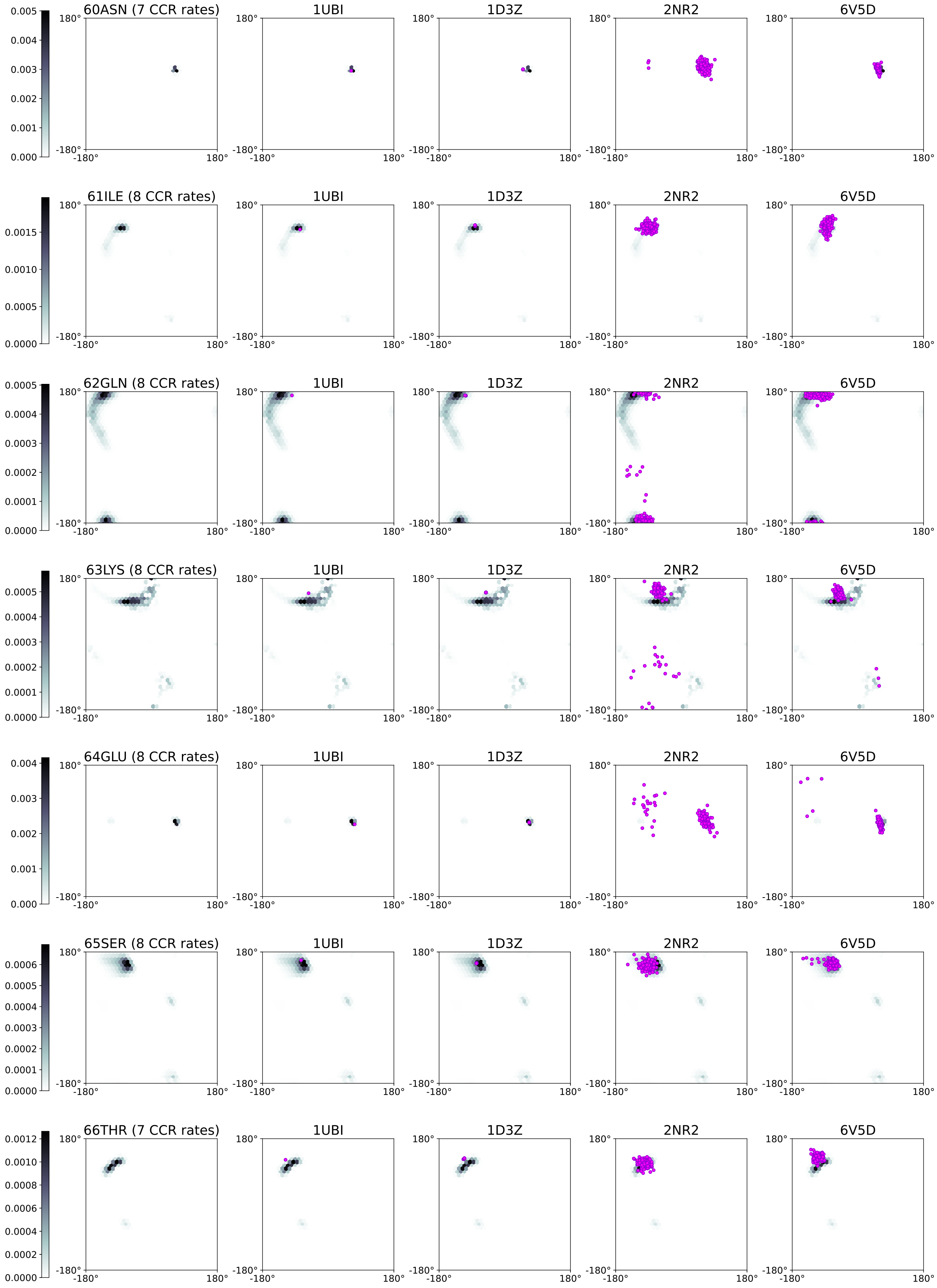

60ASN (7 CCR rates)
61ILE (8 CCR rates)
62GLN (8 CCR rates)
63LYS (8 CCR rates)
64GLU (8 CCR rates)
65SER (8 CCR rates)
66THR (7 CCR rates)
1UBI
1D3Z
2NR2
6V5D
180°
-180°

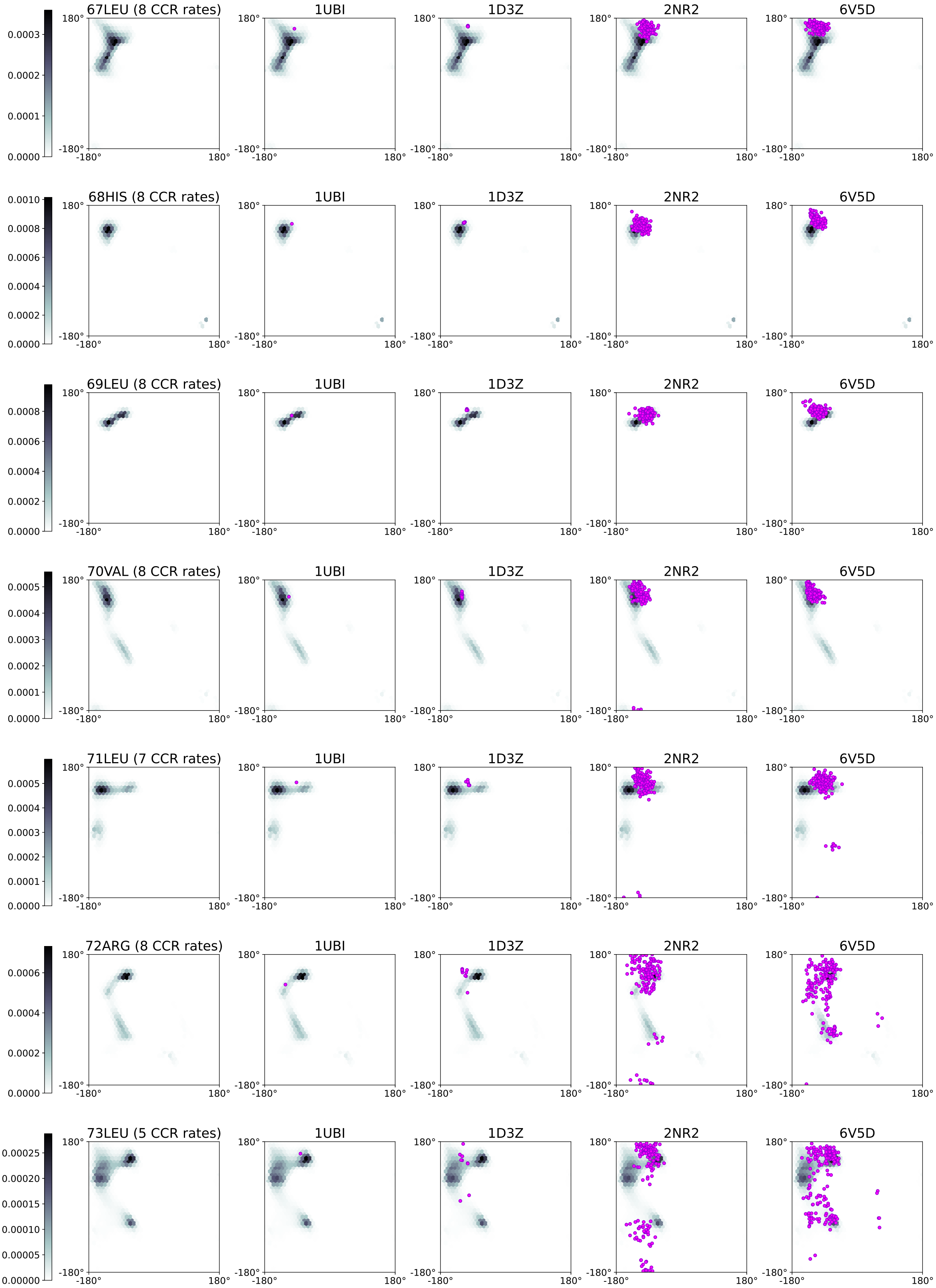

Fig. 6 Backbone dihedral angle distribution plots for all measured amino acid residues. ϕ angles are shown on the x-axes and ψ angles - on the y-axes. Each row represents a single residue. Figures in the first column show the probability densities of individual backbone conformations, as obtained from maximum entropy analysis. In columns 2 to 5, the maps are overlaid with the values of the backbone dihedral angles obtained from the ubiquitin structures deposited in the PDB database: 1UBI, 1D3Z, 2NR2, 6V5D.
